# Meta-Analysis of Genome-Wide Scans for Human Adult Stature Identifies Novel Loci and Associations with Measures of Skeletal Frame Size

**DOI:** 10.1371/journal.pgen.1000445

**Published:** 2009-04-03

**Authors:** Nicole Soranzo, Fernando Rivadeneira, Usha Chinappen-Horsley, Ida Malkina, J. Brent Richards, Naomi Hammond, Lisette Stolk, Alexandra Nica, Michael Inouye, Albert Hofman, Jonathan Stephens, Eleanor Wheeler, Pascal Arp, Rhian Gwilliam, P. Mila Jhamai, Simon Potter, Amy Chaney, Mohammed J. R. Ghori, Radhi Ravindrarajah, Sergey Ermakov, Karol Estrada, Huibert A. P. Pols, Frances M. Williams, Wendy L. McArdle, Joyce B. van Meurs, Ruth J. F. Loos, Emmanouil T. Dermitzakis, Kourosh R. Ahmadi, Deborah J. Hart, Willem H. Ouwehand, Nicholas J. Wareham, Inês Barroso, Manjinder S. Sandhu, David P. Strachan, Gregory Livshits, Timothy D. Spector, André G. Uitterlinden, Panos Deloukas

**Affiliations:** 1 Human Genetics Department, Wellcome Trust Sanger Institute, Hinxton, Cambridge, United Kingdom; 2 Department of Twin Research and Genetic Epidemiology, St. Thomas' Hospital Campus, King's College London, London, United Kingdom; 3 Department of Internal Medicine, Erasmus Medical Center, Rotterdam, The Netherlands; 4 Department of Epidemiology, Erasmus Medical Center, Rotterdam, The Netherlands; 5 Sackler Faculty of Medicine, Tel Aviv University, Tel Aviv, Israel; 6 Department of Medicine, Jewish General Hospital, McGill University, Montreal, Quebec, Canada; 7 Department of Haematology of Cambridge and NHS Blood and Transplant (NHSBT), Cambridge, United Kingdom; 8 ALSPAC Laboratory, Department of Social Medicine, University of Bristol, Bristol, United Kingdom; 9 Medical Research Council Epidemiology Unit, Institute of Metabolic Science, Addenbrooke's Hospital, Cambridge, United Kingdom; 10 Department of Public Health and Primary Care, Strangeways Research Laboratory, University of Cambridge, Cambridge, United Kingdom; 11 Division of Community Health Sciences, St. George's, University of London, London, United Kingdom; Queensland Institute of Medical Research, Australia

## Abstract

Recent genome-wide (GW) scans have identified several independent loci affecting human stature, but their contribution through the different skeletal components of height is still poorly understood. We carried out a genome-wide scan in 12,611 participants, followed by replication in an additional 7,187 individuals, and identified 17 genomic regions with GW-significant association with height. Of these, two are entirely novel (rs11809207 in *CATSPER4*, combined *P*-value = 6.1×10^−8^ and rs910316 in *TMED10*, *P*-value = 1.4×10^−7^) and two had previously been described with weak statistical support (rs10472828 in *NPR3*, *P*-value = 3×10^−7^ and rs849141 in *JAZF1*, *P*-value = 3.2×10^−11^). One locus (rs1182188 at *GNA12*) identifies the first height eQTL. We also assessed the contribution of height loci to the upper- (trunk) and lower-body (hip axis and femur) skeletal components of height. We find evidence for several loci associated with trunk length (including rs6570507 in *GPR126*, *P*-value = 4×10^−5^ and rs6817306 in *LCORL*, *P*-value = 4×10^−4^), hip axis length (including rs6830062 at *LCORL*, *P*-value = 4.8×10^−4^ and rs4911494 at *UQCC*, *P*-value = 1.9×10^−4^), and femur length (including rs710841 at *PRKG2*, *P*-value = 2.4×10^−5^ and rs10946808 at *HIST1H1D*, *P*-value = 6.4×10^−6^). Finally, we used conditional analyses to explore a possible differential contribution of the height loci to these different skeletal size measurements. In addition to validating four novel loci controlling adult stature, our study represents the first effort to assess the contribution of genetic loci to three skeletal components of height. Further statistical tests in larger numbers of individuals will be required to verify if the height loci affect height preferentially through these subcomponents of height.

## Author Summary

The first genetic association studies of adult height have confirmed a role of many common variants in influencing human height, but to date, the genetic basis of differences between different skeletal components of height have not been addressed. Here, we take advantage of recent technical and methodological advances to examine the role of common genetic variants on both height and skeletal components of height. By examining nearly 20,000 individuals from the UK and the Netherlands, we provide statistically significant evidence that 17 genomic regions are associated with height, including four novel regions. We also examine, for the first time, the association of these 17 regions with skeletal size measurements of spine, femur, and hip axis length, a measurement of hip geometry known to influence the risk of osteoporotic fractures. We find that some height loci are also associated with these skeletal components, although further statistical tests will be required to verify if these genetic variants act differentially on the individual skeletal measurements. The knowledge generated by this and other studies will not only inform the genetics of human quantitative variation, but will also lead to the potential discovery of many medically important polymorphisms.

## Introduction

Body height is determined by several biological processes that occur throughout the life of an individual and involve both normal and pathological growth. Most skeletal bones are formed by endochondral ossification, the process of replacing hyaline cartilage with bony tissue. Ossification starts shortly after gestation at the diaphysis and extends along the end of the long bones. Growth continues throughout childhood via mitotic division of the cartilage at the distal surface of the epiphysis and the epiphyseal plate, accelerates during the adolescence growth spurt and slows down in the early twenties, when the epiphyseal plate completely ossifies and peak body height is achieved. Subsequent decreases in peak body height throughout life are mainly the consequence of vertebral bone deformities such as crush fractures (osteoporosis) and cartilage degeneration (osteoarthritis).

Epidemiological studies have revealed marked differences in the growth patterns for the lower and upper portions of the body. Both trunk and lower limb length are associated with parental height, birth weight and weight at age 4 years [1]. Leg length, the principal determinant of final height attainment in adults [2], is positively associated with advantaged socio-economical circumstances and nutritional intake in childhood [1],[3],[4]. Leg length is also largely responsible for the secular increase in height in some populations [5]. Conversely, trunk length is not correlated with nutrient intake in children, and has been shown to be negatively correlated with psychophysical stress [1]. This evidence suggests that independent growth pathways might be in part responsible for final upper and lower body size.

Recent, well-powered genome-wide association (GWA) scans have already identified 43 independent loci associated with height, revealing a significant overrepresentation of genes controlling DNA replication, intracellular signalling, cell division and mesoderm and skeletal development [6]–[10]. We have carried out an independent meta-analysis of stature to assess the contribution of genetic loci to overall body height. In addition, we have investigated for the first time the contribution of these height loci to leg and trunk length using derived measurements of skeletal frame size and we have used conditional analyses to explore a possible differential contribution of the height loci to the different skeletal measurements.

## Results

### Associations with Height

To search for loci influencing adult height we analyzed genome-wide (GW) data for 299,216 SNPs from a combined sample of 12,611 adults of Caucasian origin from the UK (EPIC Norfolk, n = 3,211; TwinsUK, n = 2,224; 1958 Birth Cohort, n = 1,430) and the Netherlands (Rotterdam Study, n = 5,746) genotyped with Human300 and 550 bead arrays (Illumina) (Figure S1). We note that the EPIC Norfolk sample was also included in a recent meta-analysis for height, where the same individuals were genotyped using the 500 K Gene chip (Affymetrix) [9]. Although our discovery set was not entirely independent from the Weedon et al study, inclusion of this collection in the discovery set can provide further support to validate other genomic regions in conjunction with other sample collections. In total, 86 SNPs from 35 genomic regions reached significance with P-values<10^−5^ in the full set of 12,611 individuals; in the independent discovery set of 9,400 individuals (excluding EPIC, due to its previous use) 57 SNPs from 20 genomic regions were significant at this threshold. We selected 52 SNPs from the 35 regions for replication genotyping in an additional 4,275 samples from three population cohorts (TwinsUK, Chingford and Chuvasha). In addition, we genotyped a subset of 31 SNPs highly suggestive of true association but that did not reach GW-significance in the first replication set in additional 2,912 individuals with height data from the CBR cohort (Table 1; details of the cohorts are given in Table S1). A total of seventeen independent signals reached GW-significance (P-value≤5×10^−7^) in the combined dataset, and seven additional loci had suggestive evidence for association with height (5×10^−7^≤P-value<10^−5^, Table 1 and Table S2).

**Table 1:** Single-locus statistics for height associations of fifty-two SNPs from the thirty-four genomic regions investigated in this study.

	SNP	Chr	Pos	Band	Locus	A1/A2	MAF CEU	Discovery^2^ (n = 9,400)	Disc+EPIC^2^ (n = 12,611)	Replication^1^^,^ 2 (n = 4,275/7,187)	Combined - EPIC^2^ (n = 16,587)	Combined^3^, all cohorts (n = 19,798)		Q (d.f.)	Q P-value	I^2^	Ref 4
								P-value	P-value	P-value	P-value	P-value	Beta (SE)				
**P<5×10^−7^**	**rs11809207**	**1**	**26393727**	**1p36.11**	***CATSPER4***	**A/G**	**0.225**	**3.8×10^−6^**	**1.1×10^−5^**	**0.002**		**6.1×10^−8^**	**0.071 (0.014)**	**7.87 (7)**	**0.34**	**11**	
	**rs6763931**	**3**	**142585523**	**3q23**	***ZBTB38***	**A/G**	**0.35**	**1.8×10^−5^**	**2.2×10^−8^**	**3.1×10^−5^**	**2.3×10^−9^**	**3.1×10^−12^**	**0.070 (0.011)**	**12.21 (7)**	**0.09**	**43**	**2,3,5**
	rs6854334	4	17470308	4p15.32	*LCORL*	T/C	0.392	1.8×10^−4^	2.8×10^−7^	0.075	4.1×10^−5^	9.3×10^−8^	0.096 (0.017)	4.70 (6)	0.58	0	3,5
	rs6817306	4	17477156	4p15.32	*LCORL*	T/C	0.225	2.5×10^−4^	3.5×10^−7^	0.026	1.9×10^−5^	3.3×10^−8^	0.100 (0.018)	3.09 (6)	0.8	0	
	**rs6830062**	**4**	**17626828**	**4p15.32**	***LCORL***	**A/G**	**0.233**	**4.7×10^−5^**	**5.8×10^−8^**	**0.021**	**3.1×10^−6^**	**4.9×10^−9^**	**0.100 (0.017)**	**3.18 (6)**	**0.79**	**0**	
	**rs710841**	**4**	**82368855**	**4q21.21**	***PRKG2***	**T/C**	**0.117**	**3.4×10^−5^**	**4.3×10^−7^**	**0.016**		**2.4×10^−8^**	**0.073 (0.014)**	**9.99 (6)**	**0.13**	**40**	**2,5**
	rs2011962	4	82439348	4q21.21	*PRKG2*	T/G	0.158	6.4×10^−6^	6.2×10^−8^	0.025		6.2×10^−9^	−0.076 (0.014)	9.33 (6)	0.16	36	
	**rs10472828**	**5**	**32924575**	**5p13.3**	***NPR3***	**T/C**	**0.258**	**1.1×10^−5^**	**8.1×10^−5^**	**0.001**		**3.0×10^−7^**	**−0.058 (0.011)**	**10.63 (7)**	**0.16**	**34**	
	**rs10946808**	**6**	**26341366**	**6p22.1**	***HIST1H1D***	**A/G**	**0.432**	**3.7×10^−8^**	**4.7×10^−9^**	**2.8×10^−4^**		**5.6×10^−12^**	**0.073 (0.013)**	**1.95 (6)**	**0.92**	**0**	**2,5**
	rs9358913	6	26347383	6p22.1	*HIST1H4F*	A/G	0.15	5.7×10^−7^	4.9×10^−8^	0.047		6.8×10^−9^	0.070 (0.014)	0.53 (5)	0.99	0	
	**rs13437082**	**6**	**31462539**	**6p21.33**	***HLA-B***	**A/G**	**0.133**	**3.6×10^−8^**	**5.9×10^−8^**	**0.062**		**5.1×10^−8^**	**−0.068 (0.013)**	**8.06 (7)**	**0.33**	**13**	**5**
	rs4711269	6	31462798	6p21.33	*HLA-B*	T/C	0.155	2.2×10^−8^	1.8×10^−8^	0.158		5.5×10^−8^	−0.069 (0.013)	10.88 (7)	0.14	36	
	rs7742369	6	34273699	6p21.31	*HMGA1/C6orf106*	A/G	0.161	3.5×10^−7^	7.0×10^−8^	0.095	2.5×10^−7^	3.8×10^−8^	0.080 (0.016)	4.37 (6)	0.63	0	2,5
	**rs1776897**	**6**	**34302989**	**6p21.31**	***HMGA1/C6orf106***	**T/G**	**0.491**	**1.6×10^−8^**	**6.7×10^−9^**	**0.002**	**2.9×10^−10^**	**7.8×10^−11^**	**0.121 (0.019)**	**4.74 (7)**	**0.69**	**0**	
	rs2814993	6	34726871	6p21.31	*HMGA1/C6orf106*	A/G	0.258	1.2×10^−6^	2.5×10^−9^	0.006	4.5×10^−8^	1.4×10^−10^	0.106 (0.016)	4.24 (7)	0.75	0	
	rs12189801	6	142683299	6q24.1	*GPR126*	T/C	0.408	4.1×10^−7^	7.2×10^−7^	3.8×10^−5^		1.2×10^−10^	0.092 (0.015)	8.34 (7)	0.3	16	2,5
	**rs6570507**	**6**	**142721265**	**6q24.1**	***GPR126***	**T/C**	**0.417**	**2.1×10^−6^**	**6.4×10^−6^**	**4.9×10^−7^**		**4.4×10^−11^**	**−0.077 (0.012)**	**10.04 (6)**	**0.12**	**40**	
	**rs1182188**	**7**	**2836511**	**7p22.2**	***GNA12***	**A/G**	**0.267**	**2.2×10^−7^**	**1.1×10^−6^**	**5.9×10^−4^**		**2.8×10^−9^**	**0.066 (0.013)**	**5.72 (6)**	**0.46**	**0**	**5**
	rs1182179	7	2840174	7p22.2	*GNA12*	A/G	0.457	1.6×10^−7^	4.7×10^−7^	0.002		3.0×10^−9^	0.063 (0.013)	4.14 (6)	0.66	0	
	**rs849141**	**7**	**28151616**	**7p15.1**	***JAZF1***	**A/G**	**0.233**	**1.5×10^−5^**	**1.1×10^−6^**	**5.0×10^−6^**		**3.2×10^−11^**	**0.077 (0.012)**	**11.45 (7)**	**0.12**	**39**	
	**rs2282978**	**7**	**92102346**	**7q21.2**	***CDK6***	**A/G**	**0.075**	**6.4×10^−6^**	**3.0×10^−7^**	**0.008**	**2.6×10^−7^**	**1.2×10^−8^**	**−0.064 (0.011)**	**8.56 (7)**	**0.29**	**18**	**3,5**
	rs1480474	12	64613210	12q14.3	*HMGA2*	T/C	0.275	1.6×10^−6^	2.1×10^−9^	0.003	1.6×10^−8^	2.3×10^−11^	−0.075 (0.012)	4.18 (6)	0.65	0	1,3,5
	**rs8756**	**12**	**64646019**	**12q14.3**	***HMGA2***	**A/C**	**0.167**	**6×10^−8^**	**1.3×10^−10^**	**8.7×10^−5^**	**2.3×10^−11^**	**5.0×10^−14^**	**−0.082 (0.012)**	**5.02 (6)**	**0.54**	**0**	
	rs3118912	13	50009465	13q14.3	*DLEU7*	T/C	0.433	5.6×10^−8^	6.8×10^−10^	0.150	7.0×10^−8^	8.5×10^−10^	−0.078 (0.015)	13.06 (6)	0.04	54	3,5
	**rs3118914**	**13**	**50014902**	**13q14.3**	***DLEU7***	**A/C**	**0.392**	**3.8×10^−8^**	**2.3×10^−10^**	**0.166**	**5.9×10^−6^**	**3.9×10^−10^**	**−0.078 (0.015)**	**16.11 (6)**	**0.01**	**63**	
	rs3116607	13	50020119	13q14.3	*DLEU7*	A/C	0.292	2.3×10^−7^	3.9×10^−9^	0.189	3.2×10^−7^	5.5×10^−9^	−0.078 (0.016)	11.80 (6)	0.07	49	
	rs3118916	13	50034809	13q14.3	*DLEU7*	A/G	0.242	1.1×10^−6^	1.2×10^−8^	0.086	4.2×10^−7^	5.2×10^−9^	−0.073 (0.015)	9.38 (6)	0.15	36	
	**rs910316**	**14**	**74695795**	**14q24.3**	***TMED10***	**A/C**	**0.15**	**8.2×10^−5^**	**9.8×10^−6^**	**0.004**		**1.4×10^−7^**	**0.053 (0.011)**	**5.97 (6)**	**0.43**	**0**	
	rs2401171	15	82348680	15q25.2	*ADAMTSL3*	A/C	0.392	1.3×10^−6^	4.5×10^−7^	0.038	2.3×10^−7^	6.4×10^−8^	−0.06 (0.012)	10.04 (6)	0.12	40	3,5
	rs7183263	15	82364045	15q25.2	*ADAMTSL3*	A/C	0.417	5.7×10^−7^	1.6×10^−6^	0.006	2.6×10^−8^	4.4×10^−8^	−0.054 (0.011)	9.49 (7)	0.22	26	
	**rs4842838**	**15**	**82373128**	**15q25.2**	***ADAMTSL3***	**A/C**	**0.322**	**4.9×10^−7^**	**1.7×10^−6^**	**0.004**	**1.2×10^−8^**	**2.6×10^−8^**	**−0.055 (0.011)**	**9.59 (7)**	**0.21**	**27**	
	rs4911494	20	33435328	20q11.22	*UQCC*	A/G	0.05	7.1×10^−7^	8.1×10^−11^	3.5×10^−4^	1.0×10^−9^	1.2×10^−13^	−0.09 (0.013)	5.29 (6)	0.51	0	
	**rs6088813**	**20**	**33438595**	**20q11.22**	***UQCC***	**A/C**	**0.108**	**6.6×10^−7^**	**9.7×10^−11^**	**2.3×10^−4^**	**6.5×10^−10^**	**9.8×10^−14^**	**−0.091 (0.012)**	**5.64 (6)**	**0.46**	**0**	
**5×10^−7^<P<10^−5^**	**rs1812175**	**4**	**145794294**	**4q31.22**	***HHIP***	**A/G**	**0.492**	**0.002**	**8.5×10^−5^**	**0.016**		**3.9×10^−6^**	**−0.062 (0.016)**	**4.51 (6)**	**0.61**	**0**	2,3,5
	**rs7833986**	**8**	**57262703**	**8q12.1**	***PLAG1***	**T/C**	**0.383**	**1.6×10^−5^**	**2.2×10^−5^**	**0.427**		**1.1×10^−4^**	**−0.054 (0.014)**	**4.67 (7)**	**0.70**	**0**	2,5
	**rs7815788**	**8**	**57341574**	**8q12.1**	***PLAG1***	**A/G**	**0.392**	**4.9×10^−5^**	**5.2×10^−6^**	**0.120**		**4.8×10^−6^**	**−0.074 (0.016)**	**3.15 (7)**	**0.87**	**0**	
	**rs7871764**	**9**	**34061541**	**9p13.3**	***WDR40A***	**T/G**	**0.44**	**1.7×10^−4^**	**1.8×10^−6^**	**0.316**		**2.1×10^−6^**	**0.064 (0.013)**	**6.67 (5)**	**0.25**	**25**	
	rs7086883	10	105558830	10q24.33	*SH3PXD2A*	**T/G**	0.317	7.6×10^−4^	5.9×10^−6^	0.106		3.4×10^−6^	−0.051 (0.012)	7.74 (7)	0.36	10	
	rs3752556	16	729762	16p13.3	*NARFL*	**A/G**	0.217	5.4×10^−6^	7.9×10^−7^	0.402		8.8×10^−6^	−0.050 (0.014)	16.28 (7)	0.02	57	2
	rs12325866	17	59109706	17q23.3	*MAP3K3*	**T/C**	0.342	3.8×10^−4^	6.1×10^−6^	0.072		2.7×10^−6^	0.053 (0.012)	3.26 (7)	0.86	0	
	rs6088619	20	32875532	20q11.22	*NCOA6*	**T/C**	0.3	6.4×10^−4^	1.1×10^−5^	0.023	4.9×10^−5^	1.0×10^−6^	−0.080 (0.016)	7.02 (7)	0.43	0	3,4,5
**P>10^−5^**	rs955748	4	184452669	4q35.1	*WWC2*	**T/C**	0.342	6.1×10^−4^	4.3×10^−7^	0.941		6.6×10^−5^	−0.067 (0.013)	8.82 (7)	0.27	21	
	rs3767141	1	22088866	1p36.12	*HSPG2*	**T/C**	0.297	1.2×10^−7^	2.5×10^−5^	0.727		3.5×10^−4^	0.038 (0.011)	18.11 (7)	0.01	61	
	rs7533282	1	57406084	1p32.2	*DAB1*	**A/C**	0.1	7.6×10^−4^	7×10^−6^	0.992		3.4×10^−4^	−0.068 (0.018)	6.95 (7)	0.43	0	
	rs7596521	2	46772169	2p21	*SOCS5*	**A/G**	0.367	1.1×10^−4^	3.1×10^−6^	0.673		6.9×10^−5^	−0.043 (0.012)	8.33 (7)	0.3	16	
	rs753628	3	196040762	3q29	*FAM43A/LSG1*	**T/C**	0.258	2.9×10^−5^	9.6×10^−6^	0.741		8.6×10^−4^	0.035 (0.011)	15.73 (7)	0.03	56	
	rs2714357	6	7170994	6p24.3	*RREB1*	**A/C**	0.375	3.7×10^−5^	4.9×10^−6^	0.775		5.1×10^−4^	0.044 (0.011)	10.59 (7)	0.16	34	
	rs742106	6	15632459	6p22.3	*DTNBP1*	**A/G**	0.112	5.4×10^−6^	4.7×10^−7^	0.524		2.6×10^−4^	0.043 (0.011)	11.00 (7)	0.14	36	
	rs3131296	6	32280971	6p21.32	*NOTCH4*	**A/G**	0.367	7.7×10^−6^	3.8×10^−5^	0.097		1.8×10^−5^	0.063 (0.016)	5.92 (7)	0.55	0	
	rs1523632	7	17036493	7p21.1	*AGR2*	**T/C**	0.3	7.7×10^−6^	1.2×10^−5^	0.541		2.7×10^−5^	0.050 (0.012)	7.88 (6)	0.25	24	
	rs3125945	X	70175461	Xq13.1	*NLGN3*	**T/C**	0.367	6.8×10^−6^	3.9×10^−6^	0.656		1.4×10^−4^	0.033 (0.009)	17.37 (7)	0.02	60	
	rs1402078	X	92404317	Xq21.32	*NAP1L3*	**T/C**	0.478	5.8×10^−5^	5.8×10^−6^	0.488		3.0×10^−4^	−0.044 (0.015)	12.56 (7)	0.08	44	

1 Only SNPs with an asterisk were genotyped in the CBR collection; the sample size for the remaining SNPs is 4,274.

2 Meta-analysis P-values were calculated from study-specific best analysis P-values weighted by sample size.

3 Betas and SE for the combined sample were calculated using inverse variance meta-analysis for height values normalised to z-scores. For family-based cohorts, such values were calculated in a subset of unrelated individuals (n = 1,381 for TwinsUK discovery and n = 1,403 for TwinsUK replication).

4 1 = Weedon et al. 2007; 2 = Lettre et al. 2008; 3 = Weedon et al. 2008, 4 = Sanna et al. 2008; 5 = Gudbjartsson et al. 2008.

The SNPs having the lowest association P-value within in each region in the combined analysis are indicated in bold. For loci also described in the Weedon et al. study we report association P-values excluding the EPIC Norfolk, to account for the sample overlap with that study [9].

Two of the seventeen loci that reached GW-significant association are novel. The SNP rs11809207 is located in the third intron of the *CATSPER4* gene (combined P-value = 6.1×10^−8^). The A allele at rs11809207 was associated with an increase in height of 0.071 (95% C.I. 0.044–0.098) standard deviations, corresponding to an effect size of 0.46 cm per copy of the allele. Two nearby SNPs are in high LD with rs11809207 and have marginally higher P-values in the meta-analysis (rs2783711 near *PDIK1L*, P-value = 5.7×10^−5^, r^2^ = 0.61 in the HapMap CEU sample; and rs12069719, P-value = 3.7×10^−5^, r^2^ = 0.6; Figure S2A). The lead SNP of the second locus rs910316 (P-value = 1.4×10^−7^) is located in the first intron of *TMED10* (Figure S2O). The A allele is associated with an increase in height of 0.053 (95% C.I. 0.031–0.075) standard deviations in the combined sample, corresponding to 0.34 cm per allele copy. Two additional loci, *NPR3* and *JAZF1*, which were previously reported as weakly associated to height (P-values>10^−5^ [6]) reached GW-significance in our study (rs10472828 in *NPR3*, P-value = 3×10^−7^ and rs849141 in *JAZF1*, P-value = 3.2×10^−11^, Table 1). The remaining thirteen loci reaching GW-significance (Table 1) had been described in one or more of the recent GWA scans for stature, providing strong evidence for independent and widespread replication [6]–[10]. The Weedon et al (2008) study shared approximately 3,200 samples with our discovery cohort (EPIC cohort). Six of the eight loci discovered in both studies reached GW-significance in our replication set when EPIC was excluded from the analysis (Table 1), indicating independent replication of these loci in the remaining sample collections.

The strongest association signals in our combined set were observed at *HMGA2* (rs8756, P-value 5×10^−14^) and *UQCC* (rs6088813, P-value 9.8×10^−14^) both of which have been replicated in multiple studies [6]–[10]. The seventeen GW-significant loci explain 0.07%–0.18% of total height variance in our sample (Table 1). Finally, rs1812175 located in *HHIP* had a nominal P-value in the replication sample but did not reach GW-significance in the combined analysis (Table 1). A further six loci which did not attain nominal significance in the replication sample have suggestive evidence for association with height in the combined sample (Table 1).

To assess heterogeneity in the height associations among cohorts, we compared regression coefficients for height normalised to z-scores using the Cochran's and I^2^ statistics, finding little or no evidence for heterogeneity (Table 1). We used a similar approach to investigate gender-specific effects in height associations. We focused on the Rotterdam study, which is the largest cohort with similar numbers of males and females. We compared normalised height z-scores calculated in 3,374 females and 2,362 males from the Rotterdam Study using Cochran's and I^2^ statistics (Table S3). We observed limited evidence for gender-specific effects. The exception was *ADAMTS33* SNPs, where we detected significant heterogeneity in height associations at all three SNPs investigated (P-value = 0.002, I^2^ = 89%; Table S3).

### Associations with Skeletal Size Measurements

We tested the association of the 17 GW-significant loci with three different skeletal size measurements, namely spine length, femur and hip axis length, which provide proxies for trunk, leg and skeletal size length respectively.

We first investigated skeletal size measurements representing proxies for trunk length. We analysed 6,053 samples from three cohorts with available measurements of spine length (TwinsUK and Chuvasha) and vertebral body heights (Rotterdam Study). We combined study-specific summary statistics using z-scores and found that nine of the 17 loci were significantly associated with trunk length at the nominal level. The strongest associations with spine were at rs6570507 in *GPR126* (P-value = 4×10^−5^), rs6817306 in *LCORL* (P-value = 4×10^−4^), rs849141 in *JAZF1* (P-value = 0.001) and rs10472828 in *NPR3* (P-value = 0.0018) (Table 2).

**Table 2:** Association of validated height loci with trunk length.

SNP	Locus	A1/A2	All Samples (n = 6,053)			Rotterdam Study			
						Univariate		Height-adjusted	
			Zscore	P-value	% variance	Beta (SE)	P-value	Beta (SE)	P-value
rs11809207	*CATSPER4*	A/G	1.867	0.062	0.049	0.069 (0.036)	0.054	0.024 (0.036)	0.507
rs6763931	*ZBTB38*	A/G	2.129	0.033	0.031	0.019 (0.028)	0.49	−0.017 (0.028)	0.540
rs6854334	*LCORL*	T/C	1.648	0.099	0.100	0.118 (0.042)	0.005	0.061 (0.042)	0.142
rs6817306	*LCORL*	T/C	3.54	4×10^−4^	0.165	0.150 (0.042)	3.7×10^−4^	0.094 (0.042)	0.026
rs6830062	*LCORL*	T/C	2.86	0.004	0.212	0.162 (0.040)	5.8×10^−5^	0.091 (0.040)	0.024
rs710841	*PRKG2*	T/C	1.098	0.272	0.033	0.024 (0.032)	0.453	0.020 (0.032)	0.536
rs2011962	*PRKG2*	A/C	−0.921	0.357	0.013	−0.032 (0.032)	0.312	−0.008 (0.032)	0.794
rs10472828	*NPR3*	T/C	−3.121	0.002	0.117	−0.068 (0.028)	0.016	−0.026 (0.028)	0.366
rs10946808	*HIST1H1D*	A/G	2.657	0.008	0.111	0.007 (0.031)	0.811	−0.064 (0.031)	0.036
rs9358913	*HIST1H4F*	A/G	2.559	0.010	0.089	0.003 (0.031)	0.924	−0.057 (0.031)	0.065
rs13437082	*HLA-B*	T/C	−2.138	0.033	0.060	−0.063 (0.032)	0.052	0.011 (0.032)	0.727
rs4711269	*HLA-B*	T/C	−2.065	0.039	0.059	−0.064 (0.032)	0.047	0.011 (0.032)	0.735
rs7742369	*HMGA1/C6orf106*	A/G	−1.357	0.175	0.016	−0.039 (0.036)	0.278	−0.009 (0.036)	0.808
rs1776897	*HMGA1/C6orf106*	T/G	−2.807	0.005	0.033	−0.077 (0.048)	0.105	−0.043 (0.048)	0.373
rs2814993	*HMGA1/C6orf106*	A/G	0.923	0.356	0.013	0.050 (0.040)	0.206	−0.004 (0.040)	0.923
rs12189801	*GPR126*	T/C	2.841	0.005	0.103	0.082 (0.039)	0.038	0.076 (0.039)	0.052
rs6570507	*GPR126*	A/G	−4.109	4×10^−5^	0.220	−0.089 (0.031)	0.004	−0.058 (0.031)	0.062
rs1182188	*GNA12*	T/C	1.61	0.107	0.041	0.022 (0.032)	0.487	0.023 (0.032)	0.465
rs1182179	*GNA12*	A/G	0.502	0.615	0.036	0.021 (0.032)	0.509	0.022 (0.032)	0.486
rs849141	*JAZF1*	A/G	3.252	0.001	0.097	0.087 (0.030)	0.005	0.021 (0.031)	0.486
rs2282978	*CDK6*	T/C	−1.625	0.104	0.007	−0.014 (0.029)	0.637	0.012 (0.029)	0.694
rs1480474	*HMGA2*	A/G	1.479	0.139	0.043	0.052 (0.029)	0.070	0.003 (0.029)	0.923
rs8756	*HMGA2*	A/C	−1.484	0.138	0.033	−0.046 (0.028)	0.105	0.015 (0.028)	0.603
rs3118912	*DLEU7*	T/C	−1.505	0.132	0.101	−0.101 (0.035)	0.004	−0.007 (0.035)	0.835
rs3118914	*DLEU7*	T/G	−1.255	0.209	0.104	−0.103 (0.035)	0.003	−0.009 (0.035)	0.789
rs3116607	*DLEU7*	A/C	−2.19	0.029	0.099	−0.104 (0.037)	0.005	−0.0005 (0.037)	0.990
rs3118916	*DLEU7*	A/G	−2.443	0.015	0.073	−0.088 (0.036)	0.014	−0.005 (0.036)	0.892
rs910316	*TMED10*	A/C	−0.028	0.978	0.031	0.030 (0.028)	0.287	−0.009 (0.028)	0.765
rs2401171	*ADAMTSL3*	T/G	0.251	0.802	0.005	−0.00003 (0.028)	0.999	0.032 (0.028)	0.252
rs7183263	*ADAMTSL3*	T/G	−0.98	0.327	0.010	0.014 (0.028)	0.615	0.050 (0.028)	0.074
rs4842838	*ADAMTSL3*	T/G	1.248	0.212	0.005	−0.014 (0.028)	0.615	−0.050 (0.028)	0.074
rs4911494	*UQCC*	T/C	−1.761	0.078	0.061	−0.048 (0.029)	0.097	−0.021 (0.029)	0.477
rs6088813	*UQCC*	A/C	−1.561	0.118	0.055	−0.047 (0.029)	0.106	−0.020 (0.029)	0.494

For each locus, the meta-analysis P-value was calculated for best analysis of all cohorts with available trunk size data, namely length of spine measurements in TwinsUK and Chuvasha and sum of vertebral heights in the Rotterdam Study. Univariate and conditional analyses were carried out on a subset of 2,536 individuals of the Rotterdam Study with available vertebral height and height data.

We next tested association of the 17 confirmed height loci with hip axis length (HAL) in 2,341 individuals from the Rotterdam Study (Table 3). HAL is a highly-heritable measure of femoral geometry that measures the distance from the lateral aspect of the greater trochanter to the inner border of the pelvic rim, passing through the mid-section of the femoral neck. HAL is strongly correlated with total frame size and height [11] and represents a clinically important predictor of hip fracture independent of age and femoral neck bone mineral density [12]. Of the 17 validated height loci, seven had one or more SNPs significantly associated with HAL in the Rotterdam Study, with the strongest statistical associations observed at *LCORL* (rs6830062; P-value = 4.8×10^−4^) and *UQCC* (rs4911494; P-value = 1.9×10^−4^).

**Table 3:** Association of validated height loci with hip axis length (HAL).

SNP	Locus	A1/A2	Univariate			Height-adjusted	
			% variance	BETA (SE)	P-value	BETA (SE)	P-value
rs11809207	*CATSPER4*	A/G	0.003	−0.011 (0.038)	0.778	−0.051 (0.037)	0.174
rs6763931	*ZBTB38*	A/G	0.21	0.065 (0.029)	0.025	0.025 (0.029)	0.393
rs6854334	*LCORL*	T/C	0.31	0.118 (0.044)	0.007	0.056 (0.044)	0.195
rs6817306	*LCORL*	T/C	0.32	0.121 (0.044)	0.006	0.054 (0.044)	0.220
rs6830062	*LCORL*	T/C	0.52	0.145 (0.042)	4.8×10^−4^	0.078 (0.042)	0.062
rs710841	*PRKG2*	T/C	0.02	0.022 (0.034)	0.518	−0.010 (0.034)	0.765
rs2011962	*PRKG2*	A/C	0.07	−0.042 (0.033)	0.206	−0.008 (0.033)	0.819
rs10472828	*NPR3*	T/C	0.09	−0.043 (0.030)	0.149	−0.007 (0.030)	0.802
rs10946808	*HIST1H1D*	A/G	0.02	0.020 (0.032)	0.532	−0.015 (0.032)	0.645
rs9358913	*HIST1H4F*	A/G	0.02	0.022 (0.032)	0.492	−0.003 (0.032)	0.927
rs13437082	*HLA-B*	T/C	0.06	−0.042 (0.035)	0.226	−0.004 (0.035)	0.912
rs4711269	*HLA-B*	T/C	0.09	−0.050 (0.034)	0.146	−0.013 (0.034)	0.709
rs7742369	*HMGA1/C6orf106*	A/G	0.17	−0.074 (0.037)	0.048	−0.050 (0.037)	0.178
rs1776897	*HMGA1/C6orf106*	T/G	0.09	−0.072 (0.050)	0.151	0.014 (0.050)	0.776
rs2814993	*HMGA1/C6orf106*	A/G	0.004	−0.012 (0.040)	0.773	−0.047 (0.040)	0.236
rs12189801	*GPR126*	T/C	0.003	0.011 (0.042)	0.793	−0.039 (0.042)	0.353
rs6570507	*GPR126*	A/G	0.07	−0.043 (0.032)	0.187	0.013 (0.032)	0.689
rs1182188	*GNA12*	T/C	0.30	0.086 (0.032)	0.008	0.080 (0.032)	0.013
rs1182179	*GNA12*	A/G	0.31	0.087 (0.032)	0.007	0.082 (0.032)	0.011
rs849141	*JAZF1*	A/G	0.14	0.057 (0.032)	0.072	0.008 (0.032)	0.792
rs2282978	*CDK6*	T/C	0.15	−0.057 (0.031)	0.062	−0.040 (0.031)	0.194
rs1480474	*HMGA2*	A/G	0.26	0.074 (0.030)	0.014	0.037 (0.030)	0.214
rs8756	*HMGA2*	A/C	0.42	−0.092 (0.029)	0.002	−0.061 (0.029)	0.039
rs3118912	*DLEU7*	T/C	0.43	−0.117 (0.037)	0.002	−0.022 (0.037)	0.562
rs3118914	*DLEU7*	T/G	0.42	−0.115 (0.037)	0.002	−0.020 (0.037)	0.596
rs3116607	*DLEU7*	A/C	0.16	−0.076 (0.040)	0.055	0.030 (0.040)	0.447
rs3118916	*DLEU7*	A/G	0.28	−0.098 (0.038)	0.01	−0.015 (0.038)	0.693
rs910316	*TMED10*	A/C	0.001	0.005 (0.030)	0.865	−0.050 (0.030)	0.090
rs2401171	*ADAMTSL3*	T/G	0.15	−0.055 (0.029)	0.057	−0.033 (0.029)	0.254
rs7183263	*ADAMTSL3*	T/G	0.21	−0.065 (0.029)	0.027	−0.044 (0.029)	0.132
rs4842838	*ADAMTSL3*	T/G	0.21	0.065 (0.029)	0.027	0.044 (0.029)	0.132
rs4911494	*UQCC*	T/C	0.59	−0.112 (0.030)	1.9×10^−4^	−0.093 (0.030)	0.002
rs6088813	*UQCC*	A/C	0.57	−0.110 (0.030)	2.5×10^−4^	−0.091 (0.030)	0.002

Association of height loci with HAL in the Rotterdam Study (n = 2,341). Associations were calculated on age- and gender-adjusted (univariate) or age-, gender- and height-adjusted (height-adjusted) standardised residuals.

Finally, we investigated associations of the 17 validated height loci with measurements of lower limb length (femur) in 3,505 individuals from two cohorts (TwinsUK, N = 2,364 and Chuvasha, N = 1,141). The strongest associations with femur length were observed at rs710841 (*PRKG2*, P-value = 2.4×10^−5^) and rs10946808 (*HIST1H1D*, P-value = 6.4×10^−6^) (Table S4).

### Exploratory Conditional Analyses

We used the following qualitative approach to explore a possible differential contribution of height loci to skeletal size measurements. We selected a homogeneous set of measurements (vertebral heights and HAL in the Rotterdam Study, and femur length in TwinsUK) to avoid bias deriving from heterogeneous measurements among cohorts. Although this is expected to reduce the power to detect statistically significant associations, the magnitude of the betas is unlikely to be materially affected. Secondly, we re-calculated associations of the 17 GW-significant height loci with each skeletal size measurement as described before, only in this case we restricted the analysis to the homogeneous set of measurements. We then performed an analysis where association with each skeletal size measurement was assessed after adding height as an additional term to the linear regression model. We finally compared qualitatively the magnitude of the association, expressed as regression coefficients and SE, in the two models (univariate and height-adjusted). In cases where the locus acts prevalently through the given skeletal size measurement, we expect the regression coefficients of the height-adjusted analysis to show the least reduction compared to the original unadjusted analysis. For loci showing associations in the height-adjusted analysis, we then recalculated associations with height after adding skeletal size in the linear regression model. A null regression coefficient in this case suggests that the height association may be explained by the prevalent effect of the locus on the skeletal measurement under exam. Collectively, these conditional analyses, with relevant caveats [13], can provide some indicative information as to whether the association between relevant genetic variants and height may be mediated by specific components of height.

#### Spine

Table 2 shows that in most cases, the betas in the height-adjusted model were either close to the null (e.g. the four SNPs in *DLEU7*) or strongly reduced (e.g. for the SNPs in *HLA-B*). The *GPR126* locus (rs6570507 and rs12189801) displayed the least reduction in regression coefficients between the two models (Table 2). The regression coefficient of rs6570507 for the reversed scenario (testing association with height after adjusting for trunk size) was close to zero (−0.019 (0.031), P-value = 0.55), a result that may indicate that *GRP126* variants may contribute to height principally through trunk length elongation. *LCORL* SNPsdisplayed a similar albeit less pronounced change in the magnitude of the relevant beta coefficient, where a partial reduction in the regression coefficients (from 0.162 (0.04) to 0.091 (0.04) for rs6830062) was observed after addition of height in the model (Table 2).

#### Hip Axis Length

The results of these analyses are shown in Table 3. The *GNA12*, *HMGA2* and *UQCC* loci showed the smallest attenuation in the magnitude of the regression coefficients in the height-adjusted model. At the other extreme, the magnitudes of beta coefficients at loci such as *JAZF1* or *HLA-B* approached null in the height-adjusted model.

#### Femur length

The least reduction in regression coefficients was observed for SNPs in *ADAMTSL3* (Table S4).

### Expression Association Analysis

**Figure 1:**
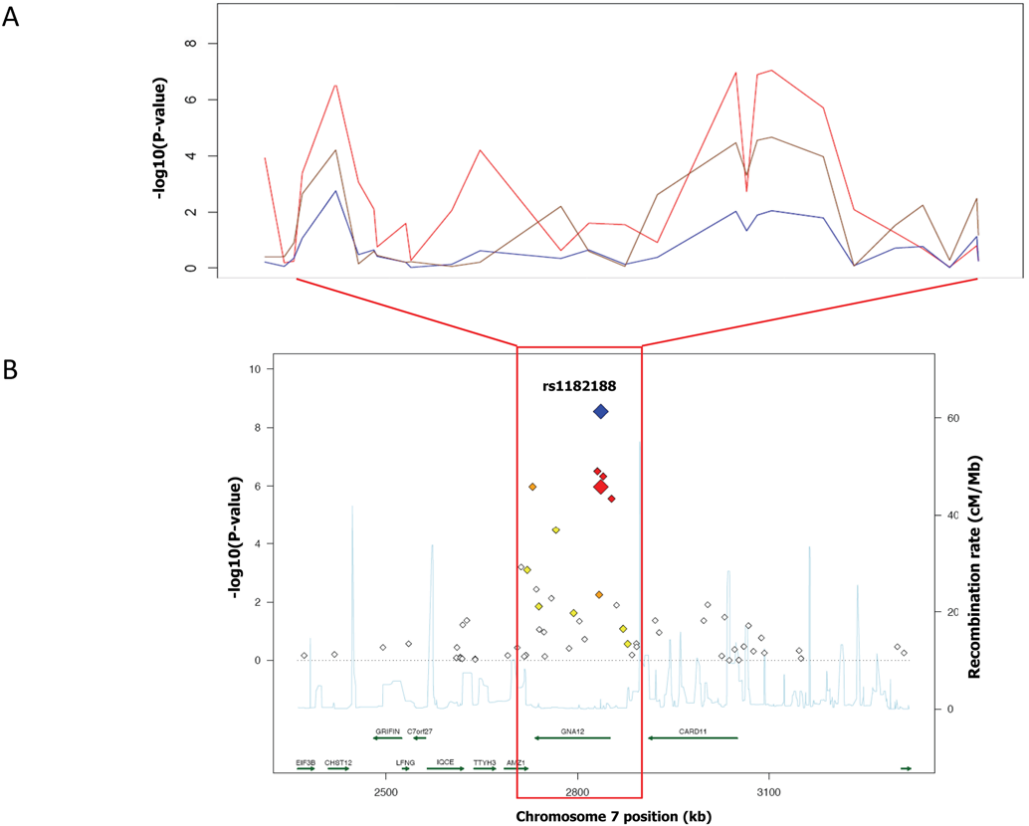
Association with gene expression in the *GNA12* region. (A) *P*-values, expressed on the −log10 scale, for associations with adult height (red) and gene expression measured at probes specific for *GNA12* (blue) and *LOC392620* (magenta) within the 370-Kb recombination interval surrounding the lead SNP rs1182188. (B) Association signal with height in the 1 MB interval surrounding the lead SNP. The red dot indicates the association *P*-value for the lead SNP rs1182188 in the discovery GWAS+EPIC sample (n = 12,611 individuals), the blue dot indicates the meta-analysis *P*-value after replication. Red diamonds indicate high LD with the lead SNP (r^2^>0.8), orange diamonds indicate moderate LD with the lead SNP (0.5<r^2^<0.8), yellow indicates markers in weak LD with the lead SNP (0.2<r^2^<0.5), white indicates either no LD with the lead SNP (r^2^<0.2), or loci where such information was not available.

Observed association signals in intergenic regions may be due to regulatory variants of nearby genes. For the 17 validated height loci (Table 1) we undertook an eQTL analysis in 2 Mb windows centered on each lead SNP (see Materials and Methods for details) using expression data from lymphoblastoid cell lines derived from individuals of the four HapMap population panels [14]. The recombination interval harboring rs1182188 contains a total of 27 SNPs tested for association with both height and expression. We tested 14 genes within ±1 MB of this interval and found significant expression association evidence (Spearman Rank correlation P-value<0.001) for 2 of them (*GNA12* and *LOC392620*) in at least one HapMap population. The rankings of the CEU expression and height association p-values at the 27 tested SNPs correlate well, suggesting a potential common functional variant underlying both the height and expression signal (*GNA12*: rho = 0.678, P-value = 0.0001; *LOC392620*; rho = 0.386, P-value = 0.047). Simulation of random significant eQTLs at this interval (as significant as the observed ones, maintaining SNP frequencies and haplotype structure) and comparison of the height-expression correlation strength to the observed data indicates that the probability of obtaining such a relationship between the two phenotypes by chance is very small (P-value = 0.0228). We detected one *cis* signal that reached significance in the eQTL analysis in a 340 Kb region of chromosome 7 centred on rs1182188. The rs1182188 SNP is located in the first intron of *GNA12* (Figure 1). *GNA12* encodes for Galpha12, a serine/threonine phosphatase modulating essential signalling pathways, including apoptosis [15],[16], regulation of the actin cytoskeleton and cadherin-mediated cell-cell adhesion [17],[18]. Even though it is known that apoptosis and cadherin-mediated signalling are involved with oncogenic effects during tumor progression, we have no clear indication how they can contribute to the determination of body height.

## Discussion

We carried out a genome-wide scan for stature in 12,611 adults of Caucasian origin and identified seventeen independent regions having GW-significant associations at the 5×10^−7^ threshold [19], and seven additional loci with suggestive evidence for association (P-values between 5×10^−7^ and 10^−5^). Two of the regions with GW-significant associations with stature are novel and were centred on an intronic SNP in *CATSPER4* (rs11809207, combined P-value = 6.1×10^−8^), and on the first intron of *TMED10* (rs910316, P-value = 1.4×10^−7^). *CATSPER4* encodes for a receptor membrane ligand ion channel with a role in acrosome reaction and male fertility [20], whereas *TMED10* (*TMP21*) encodes for a presenilin complex component with a role in vesicular protein trafficking [21]. Both these functions do not have an obvious association with height, which may be mediated by variants in nearby genes. For instance, the lead SNP in *TMED10* is in high linkage disequilibrium with three SNPs in *NEK9* displaying marginally lower association P-values in the genome-wide scan. *NEK9* is a regulator of cellular processes essential for interphase progression [22], a biological function shown to be overrepresented among height association signals [6].

We also identified two additional loci showing genome-wide statistical association with height, namely rs10472828 in *NPR3* (P-value = 3×10^−7^) and rs849141 in *JAZF1* (P-value = 3.2×10^−11^) both of which were identified with low confidence (P-value>10^−5^) in one recent GWA scan also using Illumina genome chips [6]. *NPR3* encodes for natriuretic peptide (NCP), a protein class that elicit a number of vascular, renal, and endocrine effects that are important in the maintenance of blood pressure and extracellular fluid volume [23]. *JAZF1* is a transcriptional repressor associated with a role in endometrial stromal tumors [24]. Interestingly, common variants in *JAZF1* were also recently implicated in type 2 diabetes and prostate cancer susceptibility [25],[26], in line with evidence for pleiotropic effects at many common disease loci. The remaining thirteen loci were previously described [6]–[10], confirming highly reproducible height associations despite overall small effect sizes for individual loci. Our study confirmed *HMGA2* (rs8756, P-value 5×10^−14^) and *UQCC* (rs6088813, P-value 9.8×10^−14^) as the loci with the strongest overall association with height [6]–[10].

In this study we also attempted, for the first time, to study the contribution of height loci to the components of trunk and leg length using measurements of skeletal size by directly testing associations of the 17 validated height regions with skeletal size parameters of trunk, leg and skeletal frame size, and by conducting an exploratory conditional analysis. Some intrinsic limitations of our study design and analytical approaches need to be taken into consideration in the interpretation of results for skeletal size measurements.

Firstly, although we assembled the largest dataset of this kind, the small sample size available for skeletal size associations will have affected our ability to conclusively confirm or rule out associations for some loci. However, the composite nature of height as a phenotype, and an overall higher accuracy and specificity of the skeletal measurements in assessing the contribution of genetic loci to limb length, suggests that smaller sample sizes might be sufficient to detect associations for these intermediate traits with high confidence. For example, the seventeen GW-significant height loci jointly explain approximately 5% of total variance in femur length, which is more than twice the variance in height explained by the same loci combined.

In order to increase power, we combined trunk size measurements from different cohorts that, despite being highly correlated, were not identical. Radiographic measurements of vertebral height size (Rotterdam Study) provide a more accurate measure of the skeletal component of trunk height compared to the DXA-derived measurements of the total spine (TwinsUK and Chuvasha), which include inter-vertebral disk heights and potential measurement error due to vertebral crush fractures. Measuring skeletal associations requires methods that are relatively expensive and low-throughput compared to height, making it difficult to assemble large homogeneous samples for analysis. To provide more comparable estimates of association, we focused our analyses on a subset of homogeneous standardised measurements of vertebral size and HAL in the largest available cohort, the Rotterdam Study.

To help assess whether components of skeletal height mediated the association between relevant genetic loci and height, and to examine the independency of associations between genetic loci and components of height, we conducted an exploratory conditional analysis. In this and related contexts, conditional analysis to infer conditional independence and mediation has limitations. Conditional analysis of highly correlated traits (for example, height and skeletal subcomponents of height) can lead to an attenuation of effect sizes for relevant genetic loci even when there is no underlying causal network between genetic variants and mediating traits [13]. These analyses also assume that measurement error is evenly distributed among traits. Differences in measurement error among traits can result in spurious inferences - distorting the magnitude of relevant effect sizes in conditional and unconditional analyses. The latter is also relevant to conditional analyses of genetic variants. Moreover, because of the statistical resolution required to assess differences in unconditional and conditional analyses, and the correlated variance structures of these data (testing differences in effect estimates using the same sample population) we opted to use a qualitative assessment of these interrelated associations rather than a quantitative one.

With the above caveats in mind, our results provide some interesting first insights into the potential contribution of height loci and possible differential effects on skeletal size measurements. These results should be considered exploratory and will require replication in larger cohorts to better understand their role and address potential sources of heterogeneity including the impact of measurement error. For instance, in a previous study Weedon and colleagues [10] described an association for *HMGA2* (rs1042725) with sitting height (0.2 cm increase for the C allele, 95% C.I. 0.1–0.3, P-value = 0.0002) in a cohort of approximately 2,000 children. Although this may suggest a differential effect on trunk length, in our study such association was not replicated in an adult sample of 6,509 individuals for a highly correlated SNP in the same locus (rs8756, r^2^ = 0.87 in CEU, combined P-value = 0.12). Yet, direct spine length measures in adults are likely to be more precise and specific than measures of sitting height since they are less affected by sources of measurement error that may explain such discordant results. For instance, sitting height is prone to measurement errors affected by head dimensions which are disproportionate in children (having already achieved adult head dimensions around 3 years of age, long before the pubertal growth spurt). In addition, radiographic measures of spine length will also be less affected by other artefacts arising from posture differences, age-specific growth patterns and/or possible common age-related effects of inter-vertebral disk degeneration, all of which can play a key role in this discrepancy.

The clinical relevance of the effects observed for these height loci is interesting and merits further exploration. Several loci displayed significant association with HAL, a measurement shown to vary between ethnic groups and to have substantial heritability. In addition HAL is highly correlated with long limb growth and represents an important predictor for osteoporotic fracture [12]. In our study, two intronic variants in the recombination interval containing *GDF5* and *UQCC* (rs4911494 and rs6088813) were strongly associated with HAL in the Rotterdam study (P-values = 1×10^−4^ and 1.32×10^−4^ respectively), but not with femur length (P-values = 0.83 and 0.76 respectively). *GDF5* is a member of the TGF-beta superfamily of growth factors/signalling molecules that act as regulators of cell growth and differentiation in both embryonic and adult tissues. Mutations in this gene are associated with severe skeletal malformations including acromesomelic dysplasia, Hunter-Thompson type, brachydactyly, type C and chondrodysplasia Grebe type [27]–[29]. A common functional SNP in the 5′ UTR of *GDF5* (+104T/C; rs143383) has been associated with osteoarthritis (OA), the commonest form of human arthritis characterized by degeneration of articular cartilage and bone remodelling [30],[31]. OA is also under strong genetic influence, with several shared genetic risk factors with skeletal traits including bone density, bone content, turnover and skeletal alignment [32]. The two low-stature alleles rs4911494-A and rs6088813-A were in high linkage disequilibrium with the risk T allele at rs143383 (r^2^ = 0.93), indicating a possible role of this gene in cartilage metabolism and/or bone shape and alignment in determining height.

In summary, this study extended by four the list of loci with confirmed association to adult height, which now comprises 47 independent regions. The use of fine mapping through genotype imputation and resequencing will be important for refining the association signal in each locus and for identifying the true causative variants. The potential differential effects that we observed of height loci to lower limb and trunk growth are consistent with some genes potentially acting as regulators of long-bone growth, while others appear to be specific to different bone sites or to influence cartilage growth. Further analytical and experimental approaches to assess the contribution of height loci to skeletal measurements and intermediate phenotypes will be important to understand the physiology of human growth, and may lead to the identification of genetic variants relevant to diverse musculoskeletal pathologies in humans.

**Note:** While this manuscript was in review the *JAZF1* locus was confirmed by an independent study [33].

## Materials and Methods

### Gwas Study Design

The initial discovery sample included 9,400 samples of European origin, including 1,430 British individuals (710 females and 720 males) from the British 1958 Birth Cohort, 2,224 individuals from the TwinsUK cohort (all females) and 5,746 individuals (3,374 females and 2,372 males) from a Dutch cohort (Rotterdam Study). After applying quality filters, 299,216 SNPs remained for analysis with data in at least 9,000 individuals. Further details of individual cohorts are given below and in Table S1. In addition to these three cohorts we also had available GWAS data for 3,211 samples from the EPIC Norfolk study, genotyped using the Illumina HumanHap300 (v1) SNP panel. These individuals were genotyped using the Affymetrix 500 K SNP panel in a recent height meta-analysis [9], and therefore do not constitute an independent discovery sample. Nevertheless, as the samples may provide novel loci once combined with different cohorts and a different platform, we included them in a second stage of discovery. Furthermore, these samples provide independent replication for published height signals, except than for those described in the Weedon scan [9] (Table 1). For this reason in Table 1 we provided association statistics both including and excluding the EPIC collection for all loci already described by Weedon and colleagues.

### Population Samples and Genotyping

#### TwinsUK (KCL) - discovery sample

Sample: the TwinsUK cohort (KCL, www.twinsuk.ac.uk) is an adult twin British registry shown to be representative of singleton populations and the United Kingdom population [34]. A total of 2,224 females with height phenotype were included in the discovery cohort, including 1,018 singleton individuals (754 from a MZ pair and 253 from a DZ pair and 11 of unknown zygosity) and 1,206 siblings from 603 DZ twin pairs. In order to increase power in the height analysis, we increased the sample size by considering in the analysis siblings of monozygotic twin pairs not genotyped in the GWAS. Briefly, for each MZ pair where genotype data was available (n = 754), we set the genotype data of the un-genotyped sibling to be identical to that of the genotyped twin. The phenotype was the observed phenotype for the un-genotyped twin. Corrections for sample relatedness (MZ and DZ status) using variance components were incorporated in the subsequent statistical analysis as explained in the Statistical Analysis section. The mean age of the TwinsUK cohort was 46.6 years (range 16–84). Ethics approval was obtained from the Guy's and St. Thomas' Hospital Ethics Committee. Written informed consent was obtained from every participant to the study.

Height and skeletal measurements: Standing height (cm) was measured as described in [35]. Length of spine (C4 to L4) and femur (greater trochanter to medial condyle) were taken on TwinsUK subjects (age 18–80 years) using linear pixel count (LPC) on whole body dual energy x-ray absorptiometry (DXA) imaging. LPC uses the regions of interest (ROI) sub-regional analysis mode on a Hologic QDR-4500W DXA scanner (Hologic Inc., Bedford, MA) to determine lengths of bones and has proven to be a valid and reproducible method of measuring DXA lengths with a coefficient of variation (CV%) of 1.6% [36]. Femur and spine length were strongly correlated with height (r = 0.82 and 0.59, Table S1).

Genotyping: All samples were typed with Infinium assays (Illumina, San Diego, USA) as described in Richards et al. [34]. For TwinsUK, we pooled together the normalised intensity data [37] for 2,820 Twins UK samples typed at the Duke University Genotyping Center (NC USA), Helsinki University (Finland) and the Wellcome Trust Sanger Institute. We used the Illluminus calling algorithm [38] to assign genotypes in the pooled data. No calls were assigned if an individual's most likely genotyped was called with less than a posterior probability threshold of 0.95. Validation of pooling was achieved via a visual inspection of 100 random, shared SNPs for overt batch effects. None were observed. Finally, intensity cluster plots of significant SNPs were visually inspected for over-dispersion biased no calling, and/or erroneous genotype assignment. SNPs exhibiting any of these characteristics were discarded. We excluded subjects based on the genotype data when (i) SNP call rate was <95%, (ii) heterozygosity was >37% or <33% across all SNPs, (iii) there was evidence of non-European ancestry. We corrected misclassified monozygotic and dizygotic twins based on concordance rates. At the SNP level, the QC filters applied were Hardy-Weinberg p-value≥10^−6^ (1,555 SNPs removed); MAF≥5% (9,489 SNPs removed); SNP call rate ≥95% (777 SNPs removed); 307,040 SNPs were retained for analysis.

#### The Rotterdam Study (RS) - discovery sample

Sample: Samples in this cohort were derived from the Rotterdam Study (n = 7,983), a single-center prospective population-based study of determinants of chronic disabling diseases in elderly individuals (age 55 years and over) [39],[40]. DNAs from 5,746 individuals with height information (3,374 females and 2,372 males) were genotyped using the Illumina HumanHap 550 K SNP array as part of a large population based project on genetics of complex traits and diseases financed by the Dutch government through the Netherlands Scientific Organization - Large Investments (NWO Groot; 175.010.2005.011). The mean age of the samples analysed was 68.3 years (range 55–99). Written informed consent was obtained from every participant and the study was approved by the Institutional Ethics Review Committee.

Height and skeletal measurements: Standing height (cm) measurements were attained by a trained research assistant at the baseline visit using a standard wall-mounted statiometer. Thoracolumbar spine lengths (cm) extending from T7 to L4 were calculated by summing the mean of anterior, medial and posterior vertebral heights assessed radiographically in 2550 genotyped subjects after a mean follow-up of 6.4±0.4(SD) years. X-rays were scored for the presence of vertebral fractures using the MCCloskey/Kanis method [41] and only individuals without incident or prevalent vertebral fractures were included in the analysis. HAL measurements [12] were obtained from DXA femoral scans performed after a mean follow-up of 11.1±0.6 (SD) years using a Prodigy™ fan-beam densitometer (GE-LUNAR corporation Madison, WI). Body height at baseline was strongly correlated with both follow-up measurements of spine length r = 0.78 and HAL r = 0.79.

Genotyping: Genotyping of the samples from the Rotterdam study was carried out at the Genetic Laboratory of the Department of Internal Medicine of Erasmus MC, Rotterdam, The Netherlands using the HumanHap500 V.3 Illumina SNP Array. The Beadstudio GenCall algorithm was used for data calling an quality control (QC) procedures were performed as described earlier [34]. For this study QC filters were applied to the 316,334 SNPs overlapping with the HumanHap300 set, and included Hardy-Weinberg p-value≥10^−6^ (2,229 SNPs removed); MAF≥5% (9,056 SNPs removed); SNP call rate ≥95% (4,135 SNPs removed); 306,942 SNPs overlapping with the TwinsUK and 1958 Birth Cohort data were retained for analysis.

#### 1958 Birth Cohort (58BC) - discovery sample

Sample: The British 1958 Birth Cohort is a national population sample of individuals born within a single week in 1958, and followed periodically from birth to age 44–45 years, when a DNA bank was established as a national reference series for case-control studies. The samples selected for genotyping correspond to 1502 cohort members included as population controls in the Wellcome Trust Case-Control Consortium [39]. After quality control checks for contamination, non-Caucasian identity, relatedness and low call rate (<93%), 1430 individuals (710 females and 720 males) were available with whole-genome data and were included in the meta-analysis (http://www.b58cgene.sgul.ac.uk/).

Height measurements: Standing height was measured in the home, and adjusted throughout for instrument and survey nurse (which also adjusts for any geographical variations). Z-scores were calculated on untransformed height data (cm), standardising height by gender. Field protocols, informed consent and this within-cohort genetic association analysis were approved by the South East NHS Multi-Centre Research Ethics Committee.

Genotyping: Genomic DNAs from 1958 Birth Cohort samples were genotyped at WTSI using the HumanHap500 Illumina array. Genotypes were called using the GenCall algorithm and two rounds of manual curation. The same QC filters were applied to the SNPs overlapping with HumanHap300 chip. Hardy-Weinberg p-value≥10^−6^ (404 SNPs removed); MAF≥5% (8,404 SNPs removed); SNP call rate ≥95% (10,002 SNPs removed); 309,549 SNPs were retained for analysis.

#### EPIC Norfolk - discovery sample

Sample: The European Prospective Investigation into Cancer and Nutrition study (EPIC-Norfolk) is a prospective population study of 25,663 men and women aged between 40 and 79 years, resident in Norfolk, UK. Participants are recruited from general practice registers between 1993 and 1997 and represent an ethnically homogeneous Caucasian population. Details of recruitment, anthropometric measurements, and health examinations following standardized protocols have been published [42]. All participants gave signed informed consent and The Norwich District Health Authority Ethics Committee approved the study. The EPIC Obesity cohort analysed here has been described elsewhere [43] and includes a total of 3,211 participants randomly selected from the EPIC Norfolk Study. We analysed 2,117 samples from the general EPIC cohort distribution (mean age 59 years, range 39–77), and an additional set of 1,094 cases randomly selected from the obese individuals within this cohort as having a BMI≥30 (mean age 59.4 years, range 39–76).

Height measurements: Height and weight were measured using standard anthropometric techniques. Height z-scores were calculated by standardising untransformed height by gender and age decades. The Norwich Local Research Ethics Committee granted ethical approval for the study. All participants gave written informed consent.

Genotyping: The EPIC samples were genotyped at WTSI on whole-genome amplified DNA. Genotype data calling of the EPIC cohort was carried using the Illuminus algorithm. Prior to analysis the SNPs were subject to the QC criteria used before: Hardy-Weinberg p-value≥10^−6^ (4,915 SNPs removed); MAF≥5% (11,331 SNPs removed); SNP call rate ≥95% (8,682 SNPs removed); 295,512 SNPs were retained for analysis.

#### TwinsUK - replication sample

Sample: An additional 2,294 samples (all females) from the TwinsUK cohort described previously were genotyped as part of the replication set. 174 individuals were siblings (DZ twins) of individuals genotyped in the discovery cohort. Height and skeletal measurements were obtained as described previously.

#### The Chingford Study - replication sample

Sample: The Chingford Study is a prospective population-based longitudinal cohort. The Chingford study includes 1,003 women derived from the age/sex register of a large general practice in North London, who are representative of the general UK population in terms of weight, height and smoking characteristics [44]. The study design and rationale are described elsewhere in detail [45],[46]. The women were recruited from 1987–1989 and were seen annually for radiographs and clinical examination. All subjects completed a standardized questionnaire on medical history. The Guys&St Thomas' Trust and Waltham Forest Trust ethics committees approved the study protocol.

Height measurements: Height measurements were obtained using standard anthropometric techniques.

#### Chuvasha - replication sample

Sample: Chuvashians are a Caucasian Finno-Ugric speaking population residing in the Chuvasha and Bashkortostan autonomous regions of the Russian Federation [47]. The individuals included in the study were screened for known bone diseases and risk factors for increased bone loss (such as diabetes and hyperparathyroidism) and were naïve to common medications such as hormone replacement therapy and steroid medication. Each subject signed a written consent form containing information about the study. The study was approved by the Helsinki Ethics Committee of Tel-Aviv University, Tel Aviv, Israel.

Height and skeletal measurements: Body length was measured using a statiometer and steel rulers (with 1 mm gradations) following standard anthropometric techniques [48]. Vertebral column length (spine) was calculated as the difference between suprasternal height and lower limb length, thus excluding the cervical region. Femoral length was calculated as the difference between the lower limb length and tibial height. Femur and spine length were strongly correlated with height (r = 0.83 and 0.68, Table S1).

#### Cambridge BioResource (CBR) - replication sample

Sample: The Cambridge BioResource is a collection of 4,000 pseudo-anonymised DNA samples. The collection of DNA samples from 4,000 healthy blood donors has been established by the Cambridge Biomedical Research Centre in collaboration with NHS Blood and Transplant for use in genotype-phenotype association studies.

Height measurements: Information on height (cm) self-declared and obtained by questionnaire was available for 1,912 individuals.

### Replication Genotyping

Genotyping of SNPs selected for replication was carried out using mass spectrometry (Sequenom iPLEX) at the Wellcome Trust Sanger Institute following standard procedures. Details of genotyping assays are available upon request from the authors.

### Statistical Methods

#### GW-association and stage-two analyses

Associations with z-scores for the EPIC, Rotterdam and 1958 Birth Cohort were tested using a generalised linear model (1df) assuming an additive effect for the presence of each additional minor allele. Analyses were carried out using the PLINK software package [49] (EPIC Norfolk and Rotterdam Study) or Stata v.8.1. (College Station, TX, USA) (BC58). GW-association analysis in the TwinsUK sample was carried out using a score test and variance components to account for twin status, implemented in the Merlin software package [50].

#### Meta-analyses

GW-meta-analysis statistics for height and skeletal size were obtained using a weighted z-statistics method, where weights were proportional to the square root of the number of individuals examined in each sample and selected such as the squared weights sum to be 1 [51]. Calculations were implemented in the METAL package (http://www.sph.umich.edu/csg/abecasis/Metal/). In the case of related samples the effective sample size may inflate slightly the summary statistics, although by simulation this effect has been shown to be negligible. Rather than assigning an arbitrary effect size to the sample, the true sample sizes were used for calculation.

#### Individual level analyses and quantile-quantile plots

All individual-level analyses were implemented in the R statistical package (www.r-project.org) or in StatsDirect. Quantile-quantile plots were produced in R using custom scripts based on the SNPassoc package [52].

#### Population stratification

We determined possible bias arising from population substructure by assessing for population stratification, excluding those individuals who were not of European ancestry, and by applying genomic control. For TwinsUK, stratification revealed three outliers that were removed as detailed in Richards et al. [34]. For EPIC Norfolk, we removed four outliers. There was no evidence of such effects after such exclusions, with genomic inflation factors for all four studies ≤1.02. Details of stratification of the 1958 Birth Cohort are available elsewhere [53].

#### Association with skeletal measurements

Association with skeletal measurements was tested on untransformed bone length after correcting for age (TwinsUK) and gender (Chuvasha), or analyzing sex-stratified standardized residuals adjusted for age (Rotterdam study). Associations in the TwinsUK set were tested on the combined sample of GWAS and replication genotypes, for all samples with skeletal measurements available (n = 2,375). Analyses were carried out using a score test implemented in the Merlin software. Associations in the Chuvasha sample were tested using pedigree disequilibrium test (PDT) [54]. Associations in the Rotterdam Study sample were tested using likelihood ratio and Wald tests as implemented in the PLINK software [49].

#### Calculation of effect sizes

For calculation of effect sizes for height, in order to obtain comparable estimates across all cohorts we converted height data from the family-based and twin cohorts to z-scores corrected by age and gender in a subset of unrelated individuals (n = 1,381 for the discovery set, n = 1,403 for the replication set). We then combined the summary statistics (beta and standard deviation) using inverse-variance meta-analysis and calculated the average effect size by multiplying the betas and standard errors by the average height standard deviation in our sample (6.43 cm in the combined dataset and 6.21 and 6.71 in females and males respectively).

For skeletal size measurements, we note that individual cohorts were measured using slightly different (albeit correlated) metrics for spine (total length of spine in TwinsUK and Chuvasha, and vertebral heights in the Rotterdam Study) and lower limb (femur in TwinsUK and Chuvasha, and HAL in the Rotterdam Study. To account for these differences we opted to carry out quantitative tests of heterogeneity only on one study. For trunk length, we focused on the Rotterdam study because (i) it was the largest of the three cohorts, and thus likely the best powered; (ii) the vertebral size measurements available for this cohort are a better proxy of trunk length, as they are not affected by vertebral fractures and intervertebral disk degradation; (iii) this cohort, unlike TwinsUK, contains similar numbers of males and females. For femur we used the TwinsUK collection, which represents the largest collection with available data.

#### eQTL analysis

We carried out cis-association analysis for all the 17 height signals with meta-analysis P-values<5×10^−7^ as follows. We retrieved gene expression data measured in lymphoblastoid cell lines derived from the HapMap II individuals as detailed in [14]. All genotyped HapMap II SNPs in recombination hotspot intervals containing the 17 best hits were interrogated for expression associations with genes within a ±1 MB window, using Spearman Rank correlation (SRC). Genes with at least one potential significant eQTL (SRC p-value<0.001) were further considered. Out of the total number of 342 genes that met the ±1 MB distance cutoff, we detected such significant associations with 45 genes in at least one HapMap population. For each of these genes, we then compared the rankings of the expression and height association p-values at all tested SNPs in the same hotspot interval using SRC.

In cases where the P-value rankings correlated well in the observed data (rho>0 and p-value<0.05), we evaluated the significance of the correlation in the CEU population by simulating random significant eQTLs. Expression values of each gene were shuffled 100,000 times and SNP associations recomputed. We considered a randomized interval significant if it contained at least one association with a smaller p-value than the most significant observed eQTL for that interval. For every significant randomized interval, the comparisons of the rankings of the shuffled expression and height associations were also recalculated. Significance was assigned by comparing the correlation coefficients at the significant randomized intervals with the initial observed values. After simulations and comparisons with the observed data, we identified one region with significant evidence for cis-effects.

### Web Resources

TwinsUK Cohort: http://www.twinsuk.ac.uk/

Chingford Cohort: http://www.chingfordstudy.org.uk/

1958 Birth Cohort: http://www.b58cgene.sgul.ac.uk/

Rotterdam Study: http://www.epib.nl/ergo.htm

R statistical package: http://www.r-project.org

METAL: http://www.sph.umich.edu/csg/abecasis/Metal/

## Supporting Information

Figure S1 Quantile-Quantile plots for association with height, calculated for (top row) the combined sample of males and females and for (medium row) females and (bottom row) males alone, with step-wise addition of additional cohorts (left to right): (i) TwinsUK; (ii) Rotterdam Study; (iii) 1958 Birth Cohort; (iv) EPIC Norfolk (Cohort); (v) EPIC Norfolk (High-BMI cases). The TwinsUK cohort is not represented in the male-specific analysis since the cohort contains only females.
(0.09 MB PDF)

Figure S2 Regional plot of the 17 confirmed associations with height for SNPs genotyped in the TwinsUK, Rotterdam, 1958 Birth Cohort, EPIC cohort and EPIC cases. Meta-analysis −log10 P-values are plotted as a function of genomic position (NCBI Build 36). The GWAS P-value for the lead SNP is denoted by a red diamond. A blue diamond indicates the P-value for the lead SNP in the replication sample. Proxies are indicated with diamonds of smaller size, with colours determined from their pairwise r^2^ values from HapMap CEU). Red diamonds indicate high LD with the lead SNP (r^2^>0.8), orange diamonds indicate moderate LD with the lead SNP (0.5<r^2^<0.8), yellow indicates markers in weak LD with the lead SNP (0.2<r^2^<0.5), white indicates either no LD with the lead SNP (r^2^<0.2), or loci where such information was not available.
(0.69 MB PDF)

Table S1 Distribution of height and skeletal size measurements in the cohorts included in the study.
(0.09 MB PDF)

Table S2 Calculation of effect size for height associations by cohort. Beta and SE for height values are given in z-score units for each copy of A1 allele. For family-based cohorts (TwinsUK Discovery (KCL) and replication (KCL_RP) and Chuvasha) height z-scores were calculated in a subset of unrelated individuals.
(0.15 MB PDF)

Table S3 Gender-specific associations at the 17 validated height loci.
(0.11 MB PDF)

Table S4 Association of height loci with femur length. For each locus, the meta-analysis P-value was calculated for best analysis on two cohorts (TwinsUK, N = 2,364 and Chuvasha, N = 1,141). Univariate and conditional analyses were carried out on a subset of unrelated TwinsUK samples with available height and femur length.
(0.12 MB PDF)
