# Supplementary material for: Meta-Analysis of Genome-Wide Scans for Human Adult Stature Identifies Novel Loci and Associations with Measures of Skeletal Frame Size
Source: PLoS Genet. 2009 Apr 3;5(4):e1000445. doi: 10.1371/journal.pgen.1000445 (PMC2661236; doi:10.1371/journal.pgen.1000445)

Figure S1. Quantile-Quantile plots for association with height, calculated for (top row) the combined sample of males and females and for (medium row) females and (bottom row) males alone, with step-wise addition of additional cohorts (left to righ): (i) TwinsUK; (ii) Rotterdam Study; (iii) 1958 Birth Cohort; (iv) EPIC Norfolk (Cohort); (v) EPIC Norfolk (High-BMI cases). The TwinsUK cohort is not represented in the male-specific analysis since the cohort contains only females.

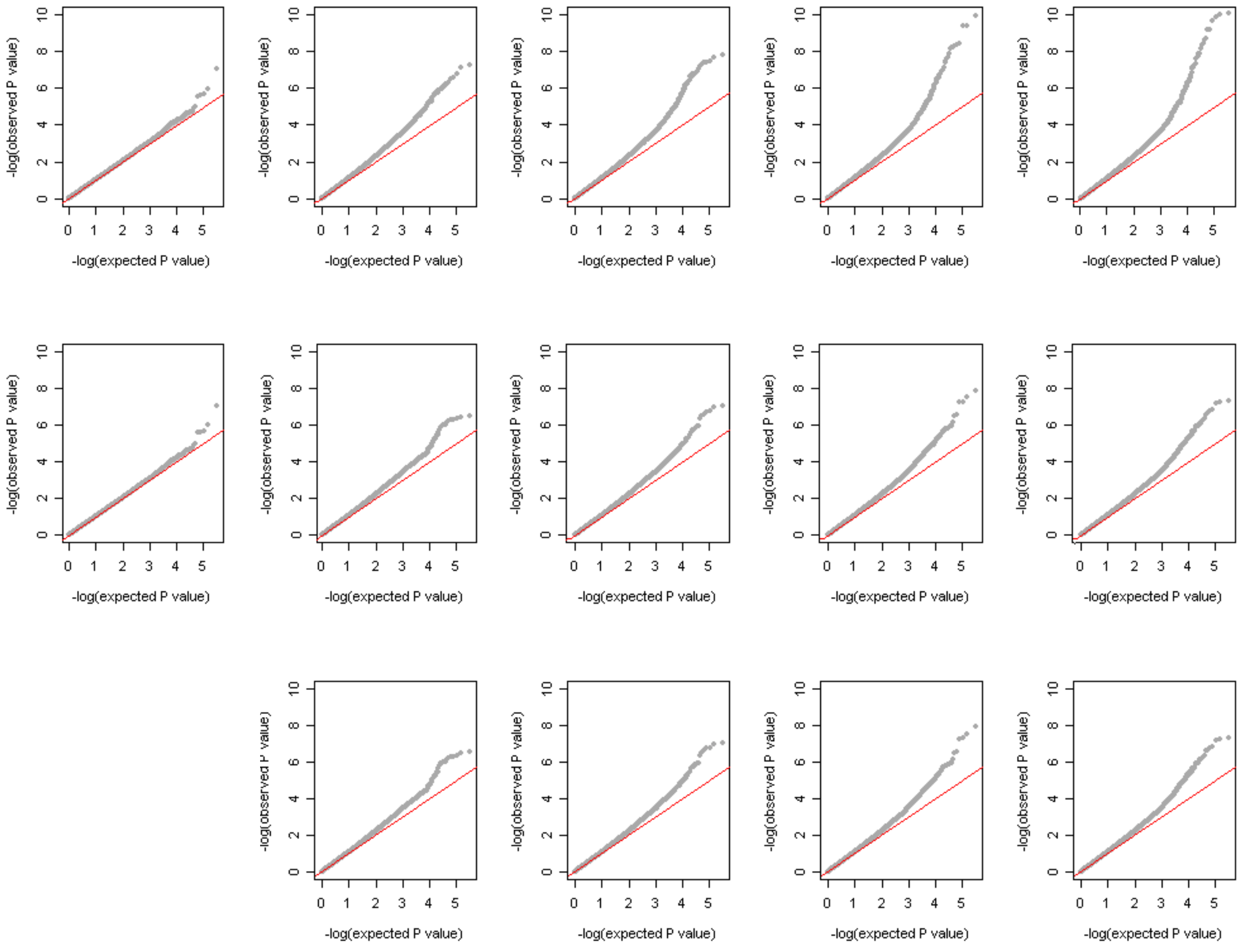

Supplement: Figure S1 — Quantile-Quantile plots for association with height, calculated for (top row) the combined sample of males and females and for (medium row) females and (bottom row) males alone, with step-wise addition of additional cohorts (left to right): (i) TwinsUK; (ii) Rotterdam Study; (iii) 1958 Birth Cohort; (iv) EPIC Norfolk (Cohort); (v) EPIC Norfolk (High-BMI cases). The TwinsUK cohort is not represented in the male-specific analysis since the cohort contains only females. (0.09 MB PDF) [file pgen.1000445.s001.pdf]
