# Supplementary material for: Meta-Analysis of Genome-Wide Scans for Human Adult Stature Identifies Novel Loci and Associations with Measures of Skeletal Frame Size
Source: PLoS Genet. 2009 Apr 3;5(4):e1000445. doi: 10.1371/journal.pgen.1000445 (PMC2661236; doi:10.1371/journal.pgen.1000445)
Supplement: Figure S2 — Regional plot of the 17 confirmed associations with height for SNPs genotyped in the TwinsUK, Rotterdam, 1958 Birth Cohort, EPIC cohort and EPIC cases. Meta-analysis −log10 P-values are plotted as a function of genomic position (NCBI Build 36). The GWAS P-value for the lead SNP is denoted by a red diamond. A blue diamond indicates the P-value for the lead SNP in the replication sample. Proxies are indicated with diamonds of smaller size, with colours determined from their pairwise r2 values from HapMap CEU). Red diamonds indicate high LD with the lead SNP (r2>0.8), orange diamonds indicate moderate LD with the lead SNP (0.5<r2<0.8), yellow indicates markers in weak LD with the lead SNP (0.2<r2<0.5), white indicates either no LD with the lead SNP (r2<0.2), or loci where such information was not available. (0.69 MB PDF) [file pgen.1000445.s002.pdf]

Figure S2: Regional plots of the 17 confirmed associations with height for SNPs genotyped in the TwinsUK, Rotterdam, 1958Birth Cohort, EPIC cohort and EPIC cases. Meta-analysis -log10 P-values are plotted as a function of genomic position (NCBI Build 36). The GWAS P-value for the lead SNP is denoted by a red diamond. A blue diamond indicates the P-value for the lead SNP in the replication sample. Proxies are indicated with diamonds of smaller size, with colours determined from their pairwise r^2 values from HapMap CEU). Red diamonds indicate high LD with the lead SNP ($r^2>0.8$), orange diamonds indicate moderate LD with the lead SNP ($0.5<r^2<0.8$), yellow indicates markers in weak LD with the lead SNP ($0.2<r^2<0.5$), white indicates either no LD with the lead SNP ($r^2<0.2$), or loci where such information was not available.

(A) *CATSPER4*

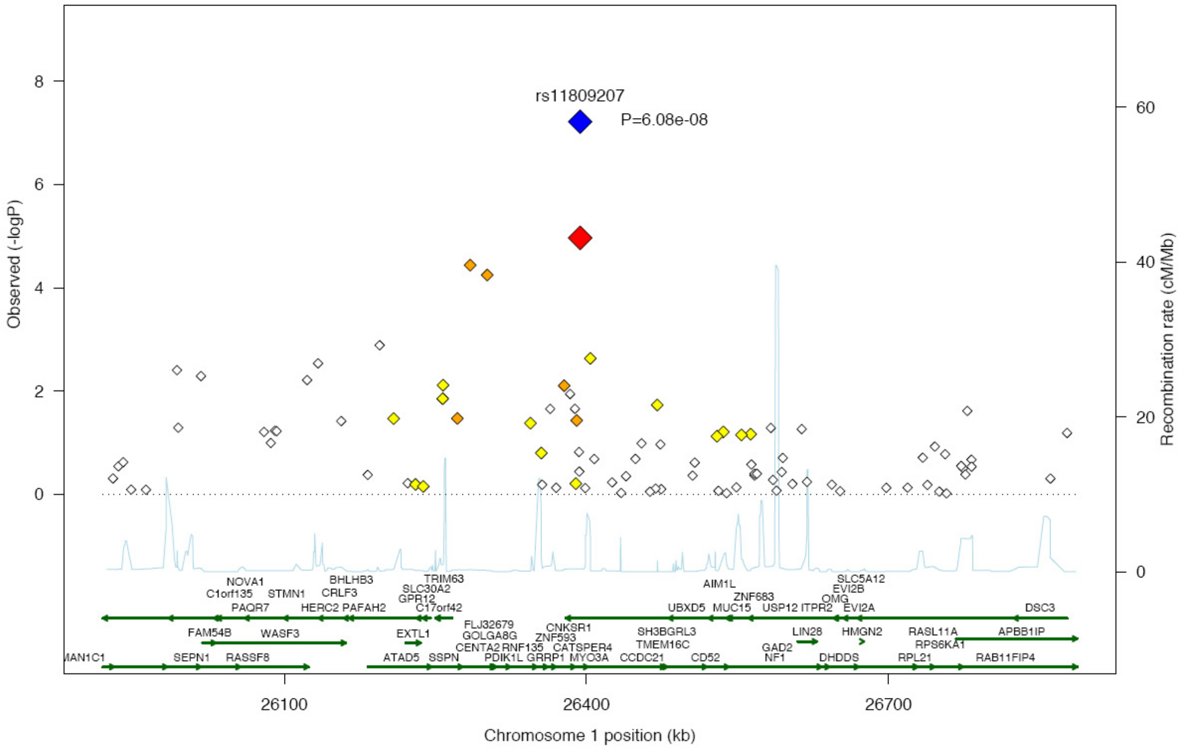

(B) *ZBTB38*

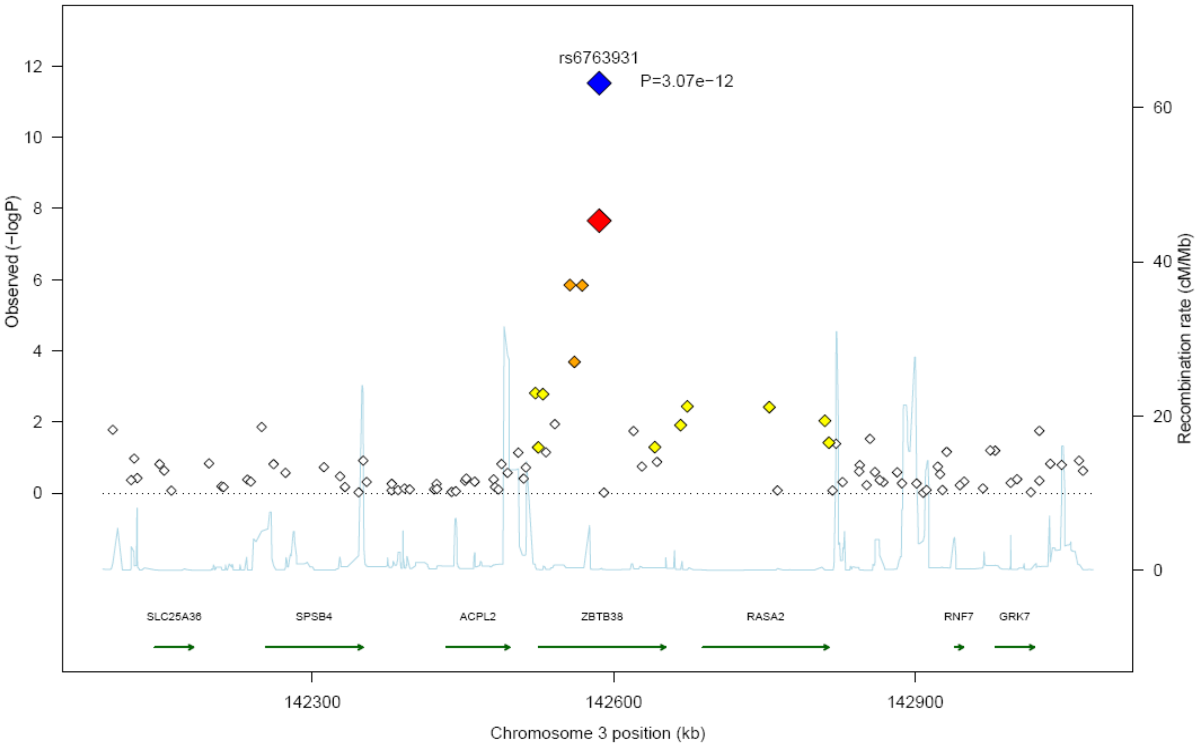

rs6763931
P=3.07e−12
Observed (−logP)
Recombination rate (cM/Mb)
SLC25A36
SPSB4
ACPL2
ZBTB38
RASA2
RNF7
GRK7
142300
142600
142900
Chromosome 3 position (kb)

(C) *LCORL*

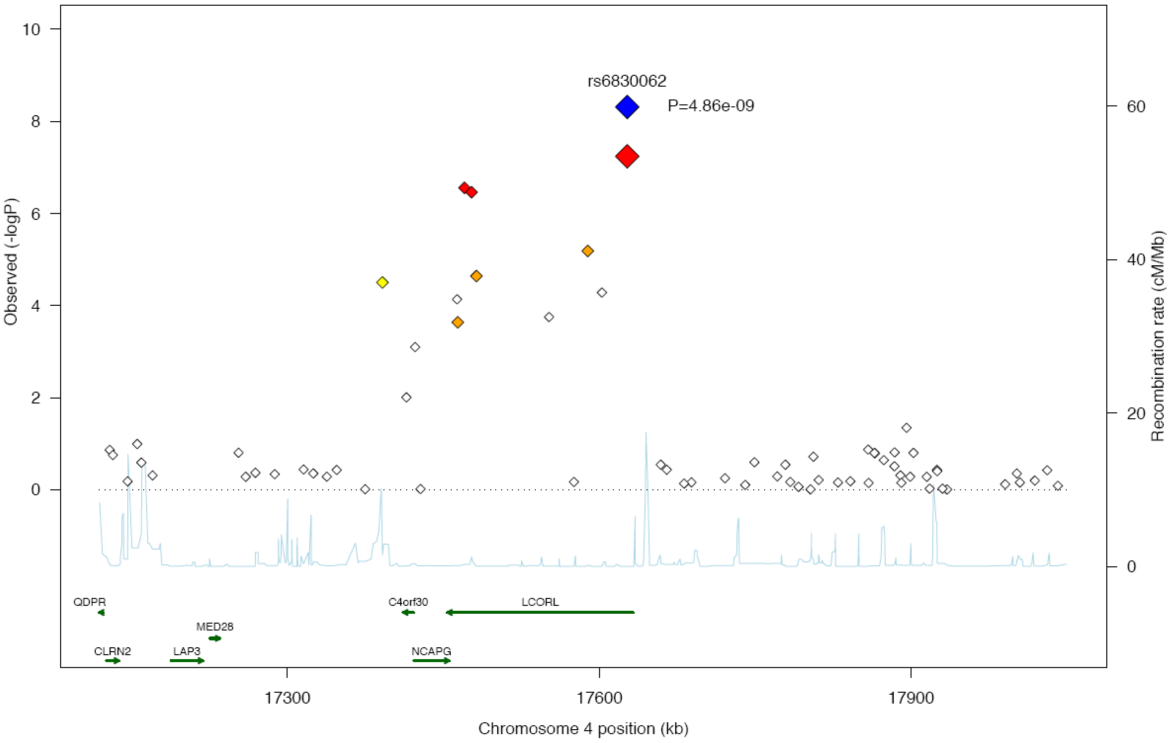
rs6830062
P=4.86e-09
Observed (-logP)
Recombination rate (cM/Mb)
Chromosome 4 position (kb)
17300
17600
17900
QDPR
CLRN2
LAP3
MED28
C4orf30
NCAPG
LCORL

(D) ***PRKG2***

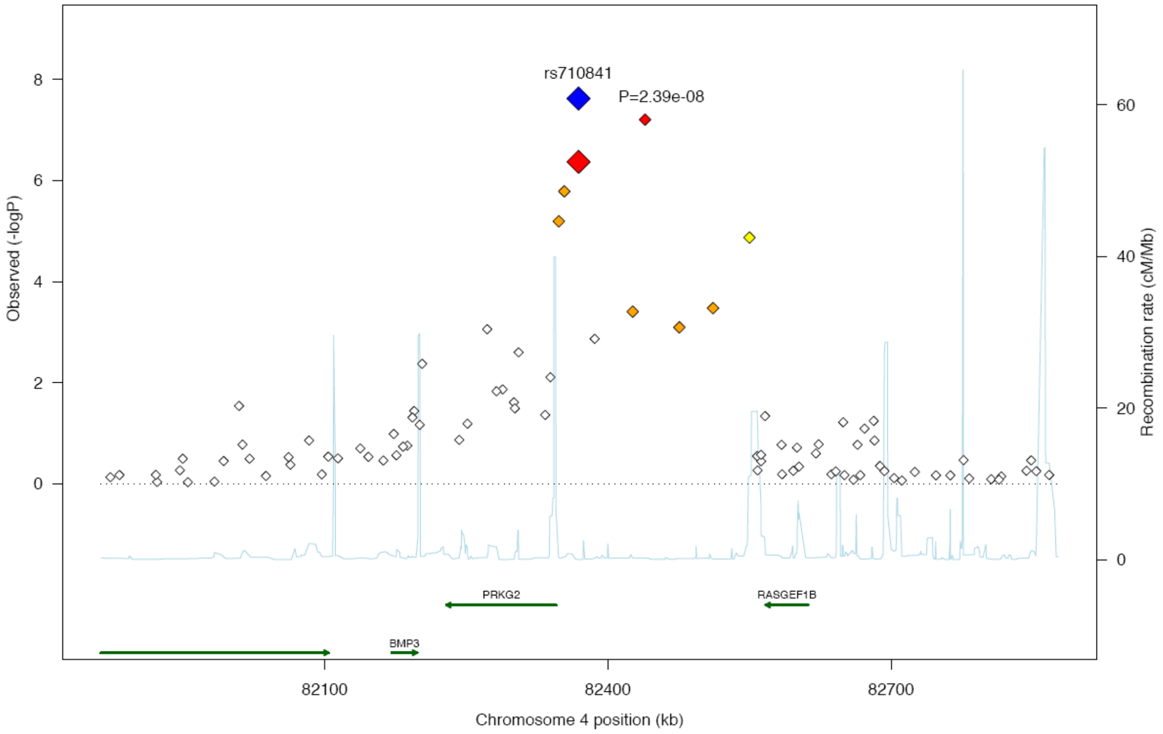

rs710841
P=2.39e-08
Observed (-logP)
Recombination rate (cM/Mb)
PRKG2
RASGEF1B
BMP3
82100
82400
82700
Chromosome 4 position (kb)

(E) ***NPR3***

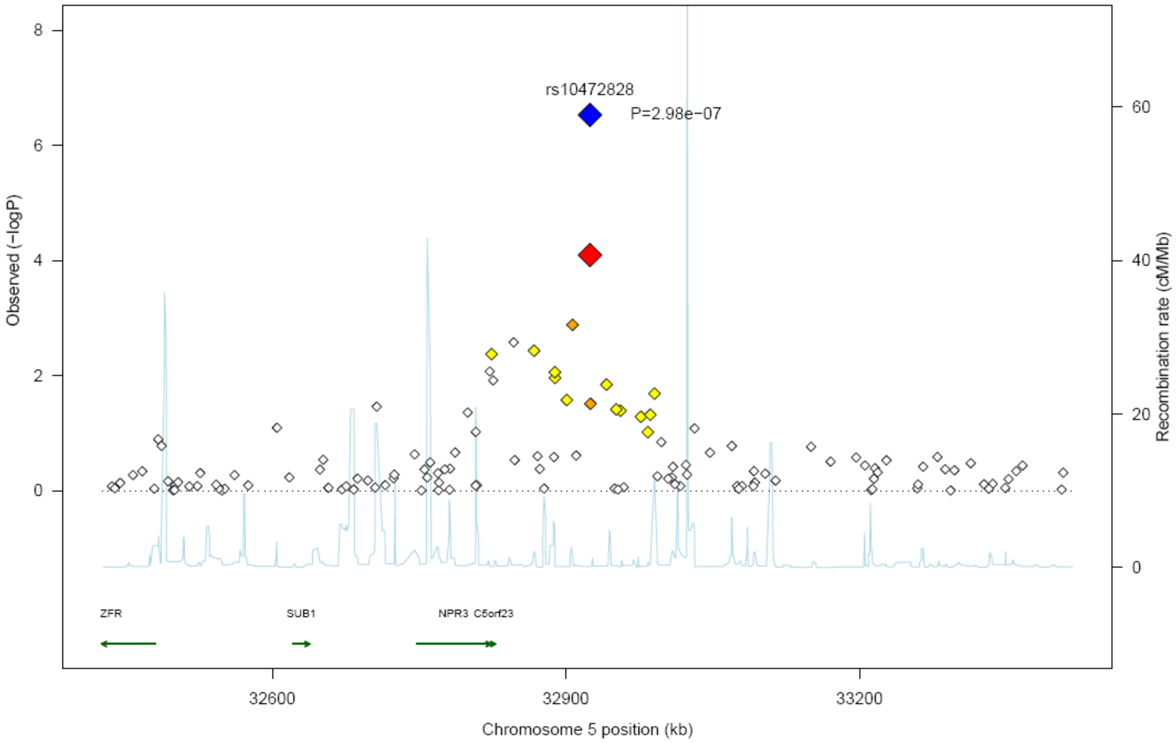

rs10472828
P=2.98e−07
Observed (−logP)
Recombination rate (cM/Mb)
ZFR
SUB1
NPR3 C5orf23
32600
32900
33200
Chromosome 5 position (kb)

(F) *HIST1H1D*

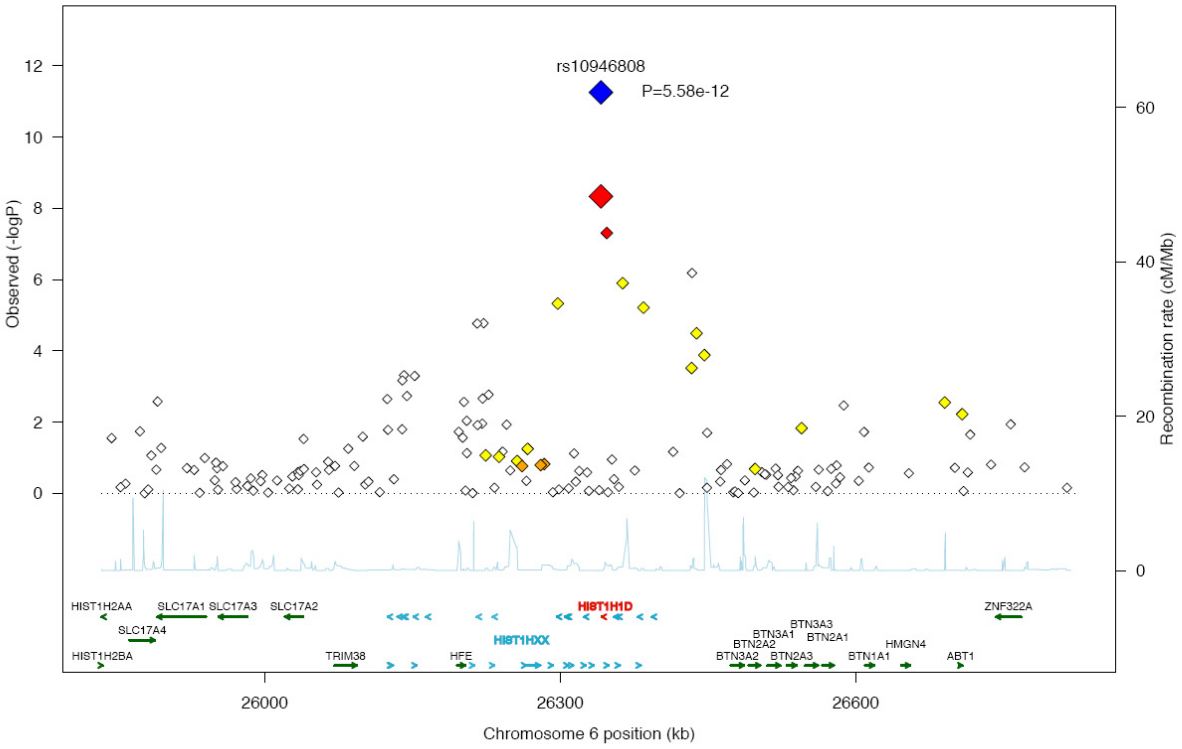

rs10946808
P=5.58e-12
Observed (-logP)
Recombination rate (cM/Mb)
HIST1H2AA
SLC17A1
SLC17A3
SLC17A2
HIST1H1D
ZNF322A
SLC17A4
HIST1HXX
BTN3A3
BTN3A1
BTN2A1
BTN2A2
HMGN4
HIST1H2BA
TRIM38
HFE
BTN3A2
BTN2A3
BTN1A1
ABT1
26000
26300
26600
Chromosome 6 position (kb)

(G) ***HLA-B***

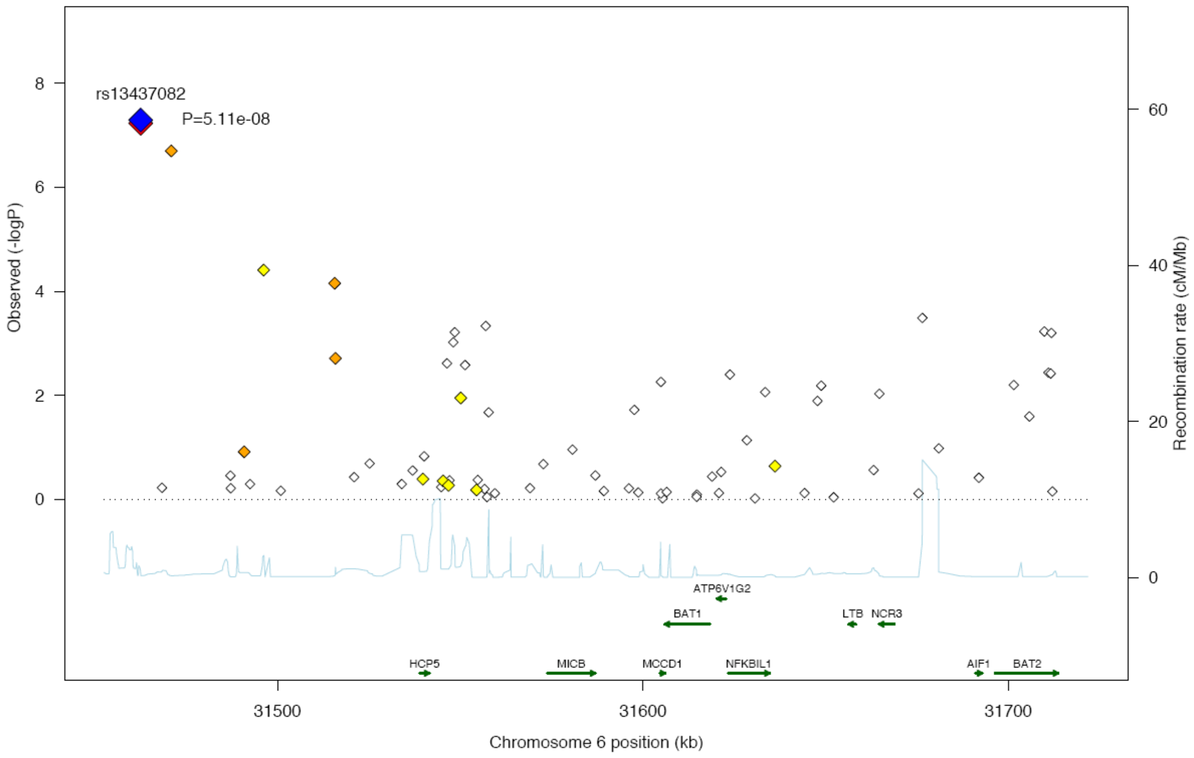

rs13437082
P=5.11e-08
Observed (-logP)
Recombination rate (cM/Mb)
Chromosome 6 position (kb)
0
2
4
6
8
0
20
40
60
31500
31600
31700
ATP6V1G2
BAT1
LTB
NCR3
HCP5
MICB
MCCD1
NFKBIL1
AIF1
BAT2

(H) ***HMGA1/C6orf106***

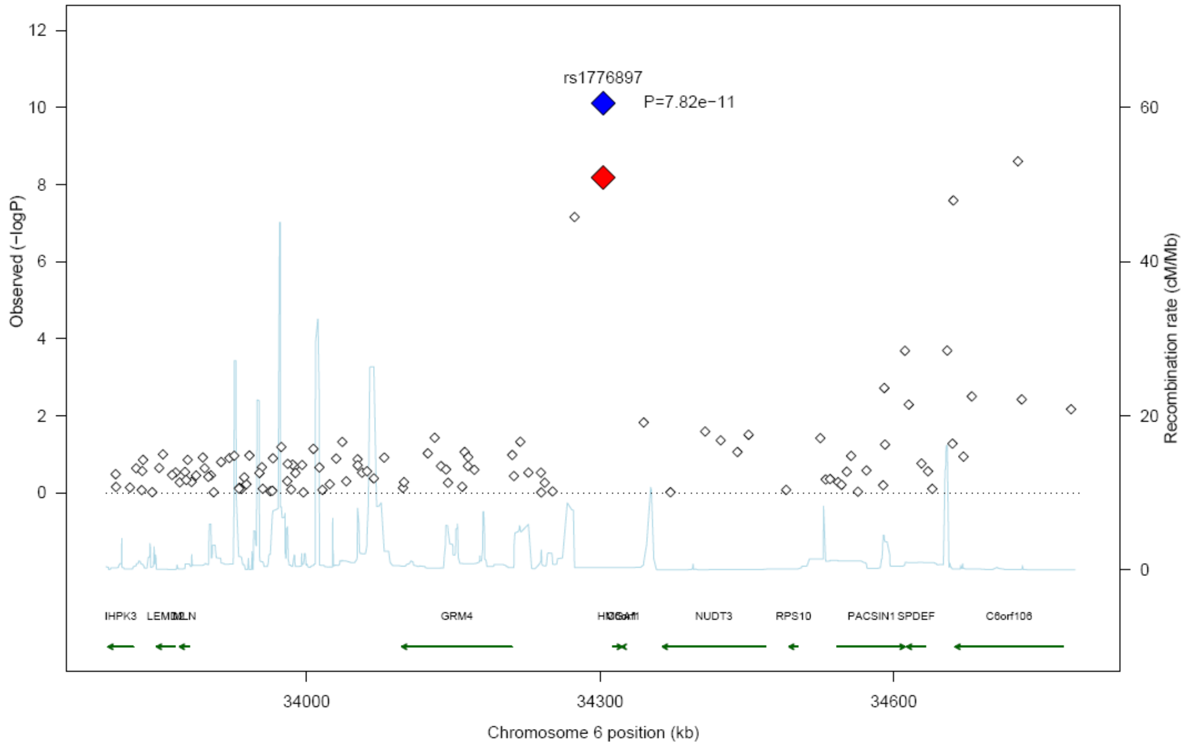

(I) ***GPR126***

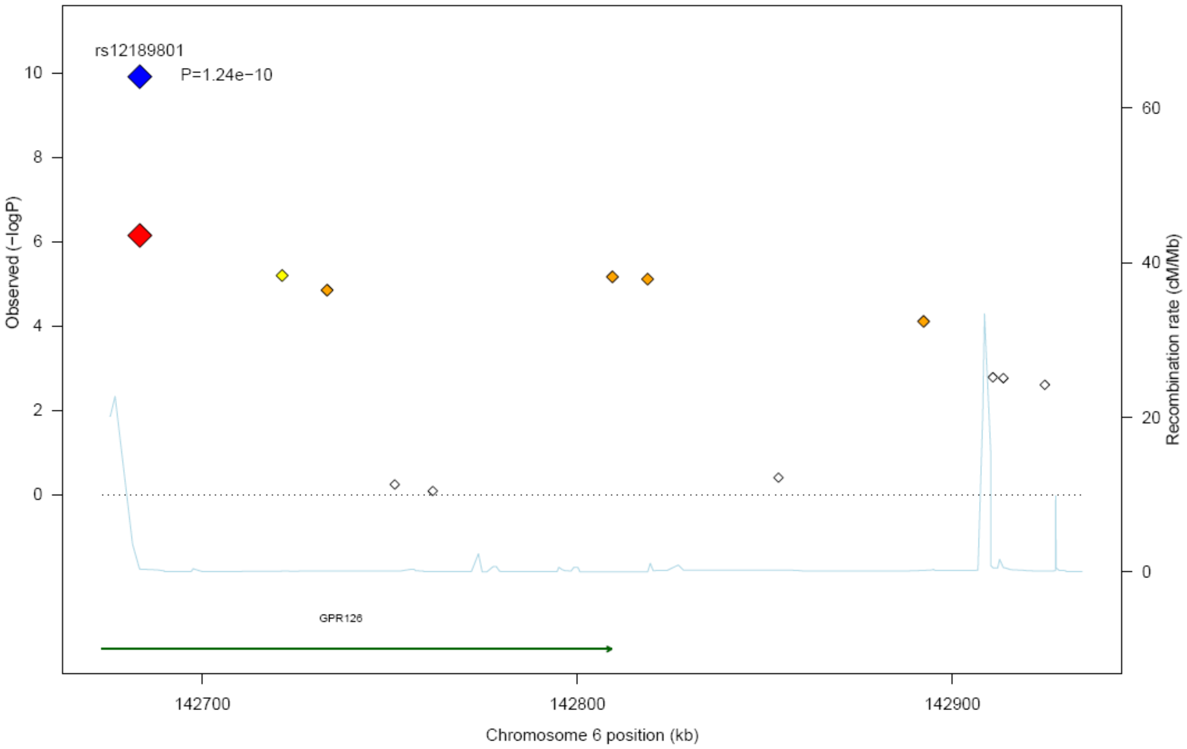
rs12189801
P=1.24e−10
Observed (−logP)
10
8
6
4
2
0
60
40
20
0
Recombination rate (cM/Mb)
GPR126
142700
142800
142900
Chromosome 6 position (kb)

(J) *GNA12*

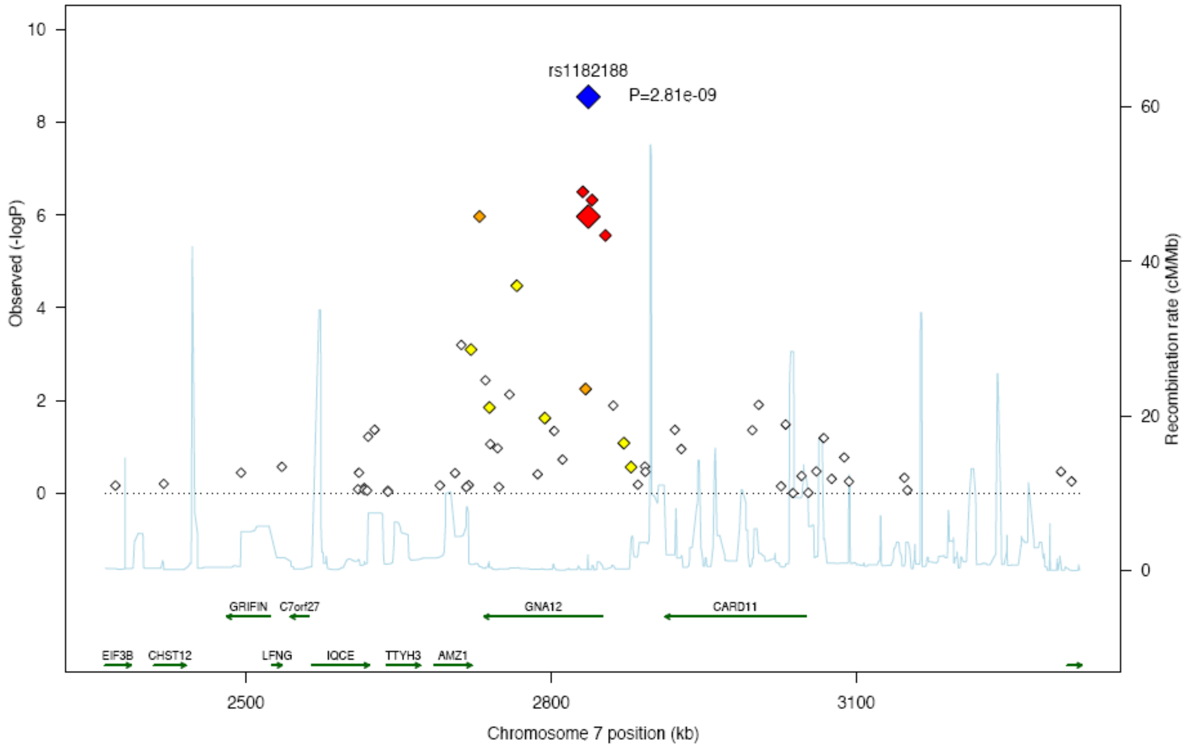

rs1182188
P=2.81e-09
10
8
6
4
2
0
Observed (-logP)
60
40
20
0
Recombination rate (cM/Mb)
GRIFIN
C7orf27
GNA12
CARD11
EIF3B
CHST12
LFNG
IQCE
TTYH3
AMZ1
2500
2800
3100
Chromosome 7 position (kb)

(K) ***JAZF1***

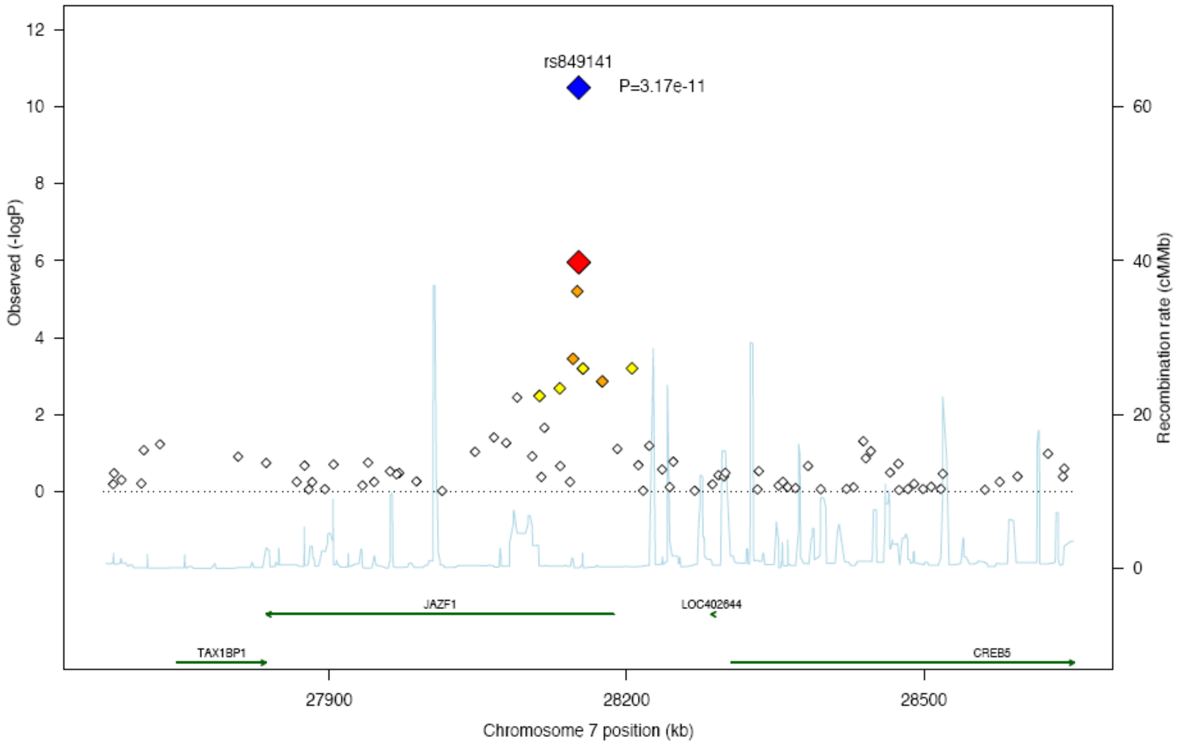
rs849141
P=3.17e-11
Observed (-logP)
Recombination rate (cM/Mb)
JAZF1
LOC402644
TAX1BP1
CREB5
27900
28200
28500
Chromosome 7 position (kb)

(L) ***CDK6***

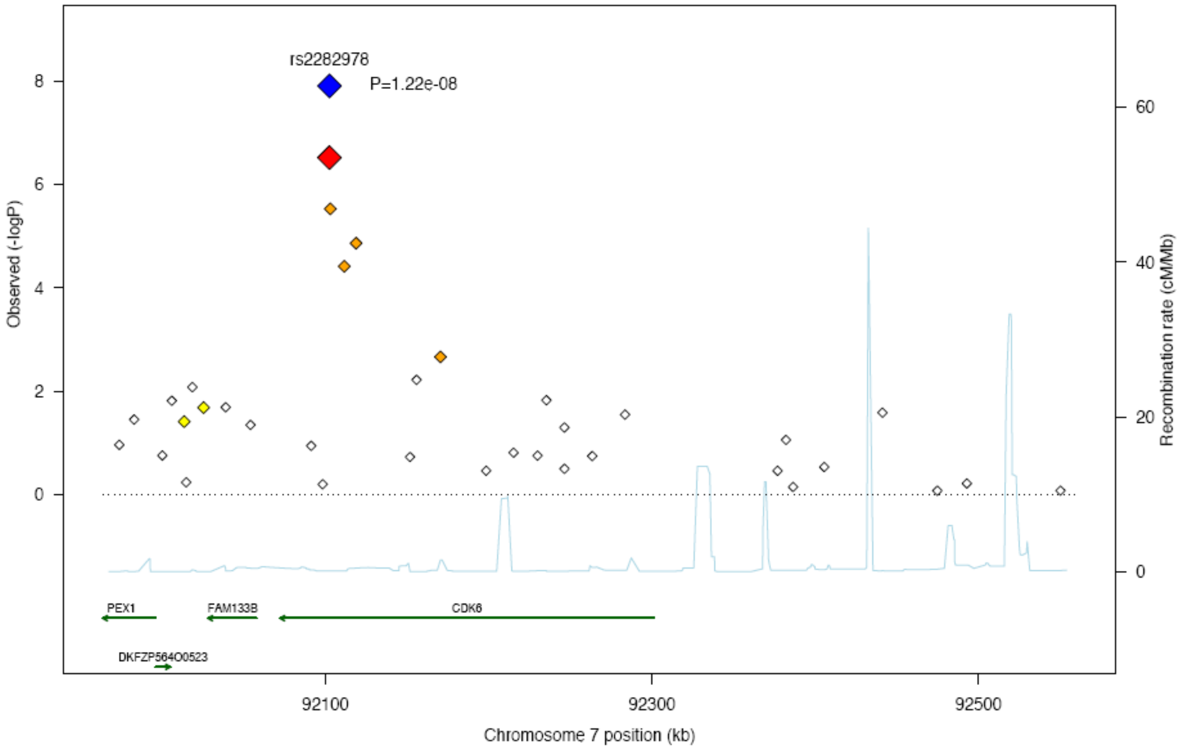

rs2282978
P=1.22e-08
Observed (-logP)
Recombination rate (cM/Mb)
0
2
4
6
8
0
20
40
60
PEX1
FAM133B
CDK6
DKFZP564O0523
92100
92300
92500
Chromosome 7 position (kb)

(M) ***HMGA2***

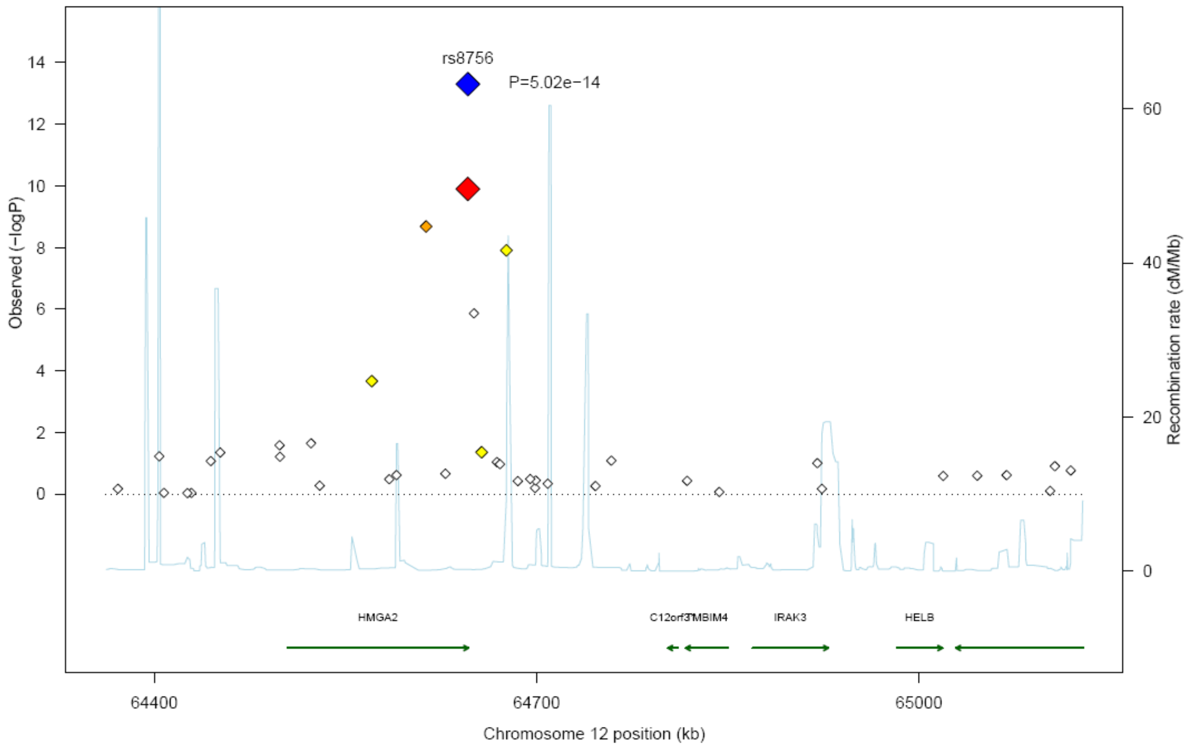

rs8756
P=5.02e−14
Observed (−logP)
14
12
10
8
6
4
2
0
Recombination rate (cM/Mb)
60
40
20
0
HMGA2
C12orf3
TMBIM4
IRAK3
HELB
64400
64700
65000
Chromosome 12 position (kb)

(N) ***DLEU7***

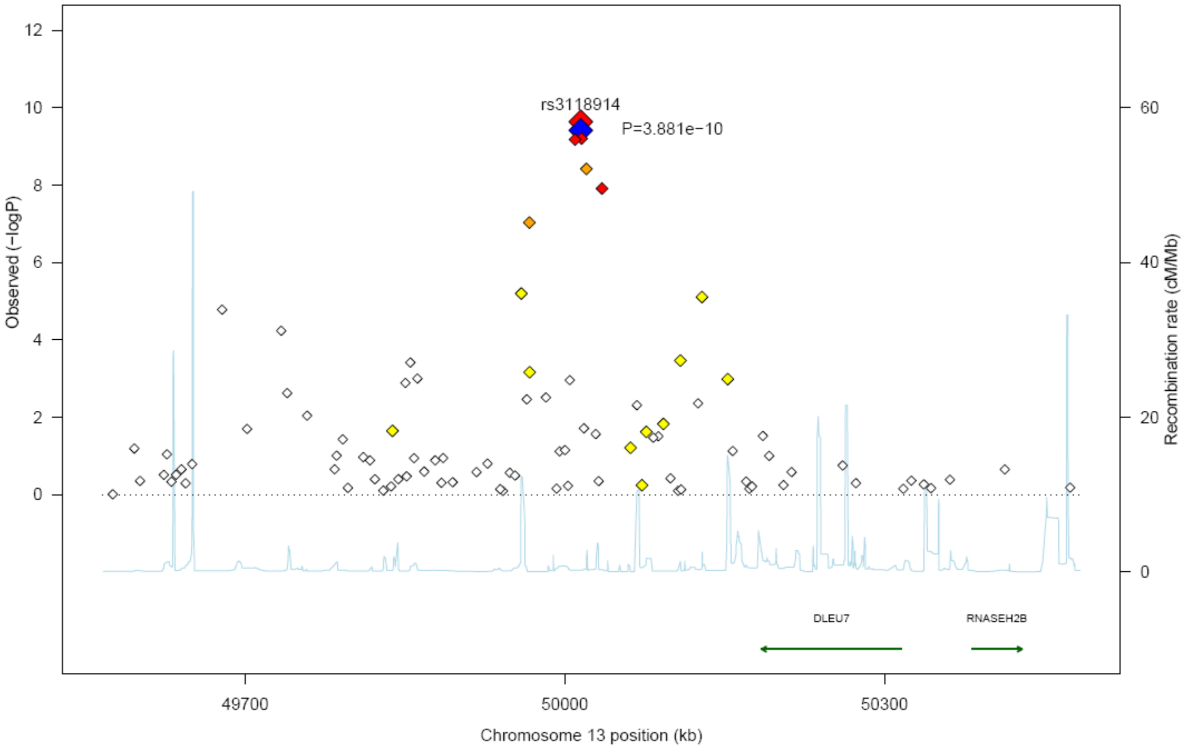

rs3118914
P=3.881e−10
Observed (−logP)
Recombination rate (cM/Mb)
DLEU7
RNASEH2B
49700
50000
50300
Chromosome 13 position (kb)

(O) ***TMED10***

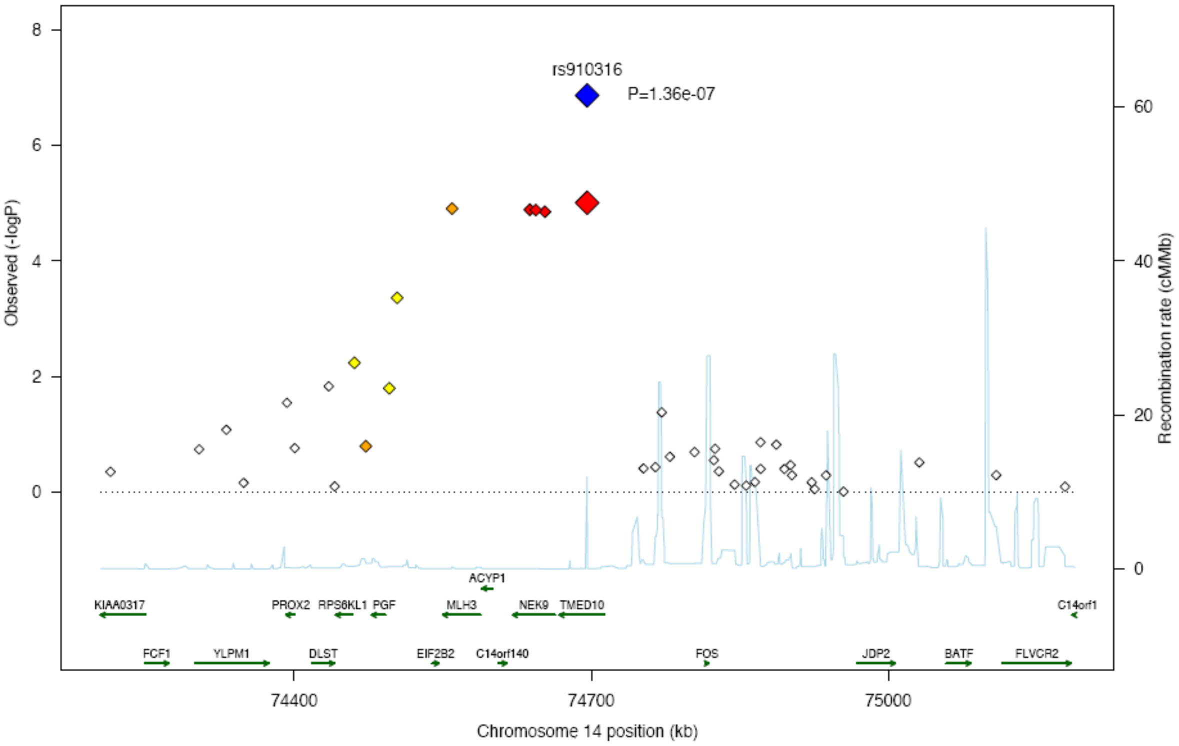

rs910316
P=1.36e-07
Observed (-logP)
Recombination rate (cM/Mb)
Chromosome 14 position (kb)
74400
74700
75000
ACYP1
KIAA0317
PROX2
RPS6KL1
PGF
MLH3
NEK9
TMED10
C14orf1
FCF1
YLPM1
DLST
EIF2B2
C14orf140
FOS
JDP2
BATF
FLVCR2

(P) ***ADAMTSL3***

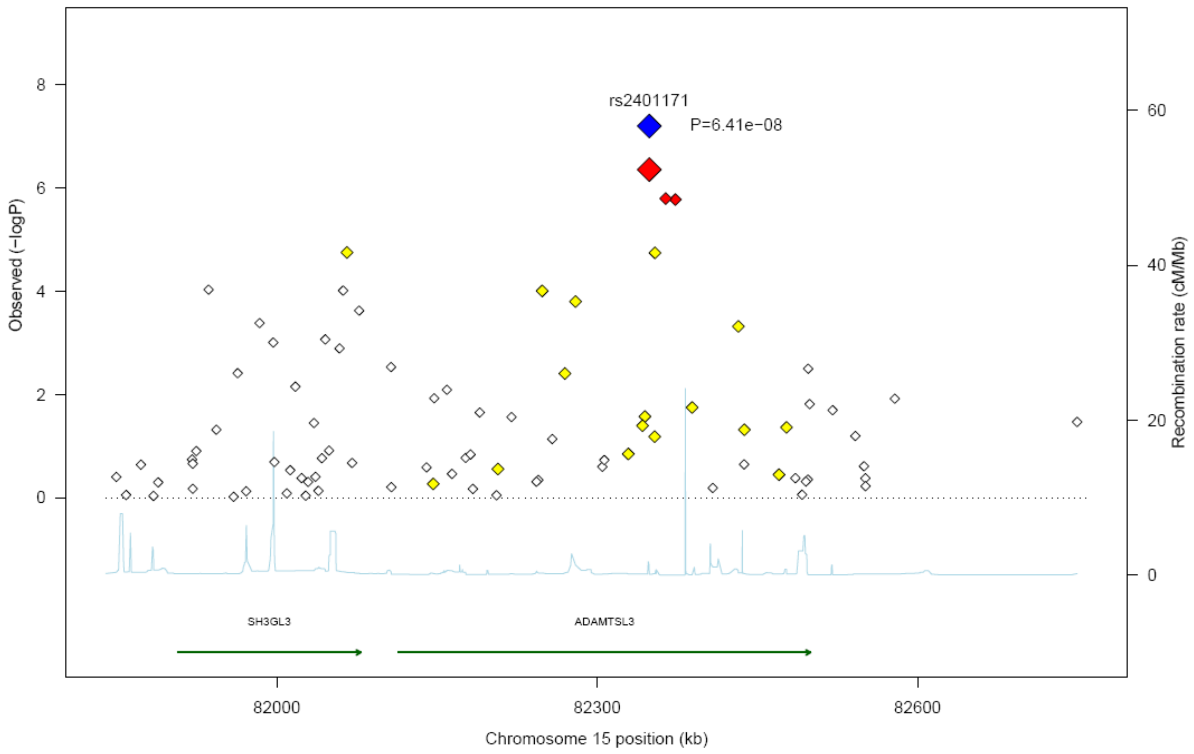

rs2401171
P=6.41e−08
Observed (−logP)
Recombination rate (cM/Mb)
8
6
4
2
0
60
40
20
0
SH3GL3
ADAMTSL3
82000
82300
82600
Chromosome 15 position (kb)

(Q) ***UQCC***

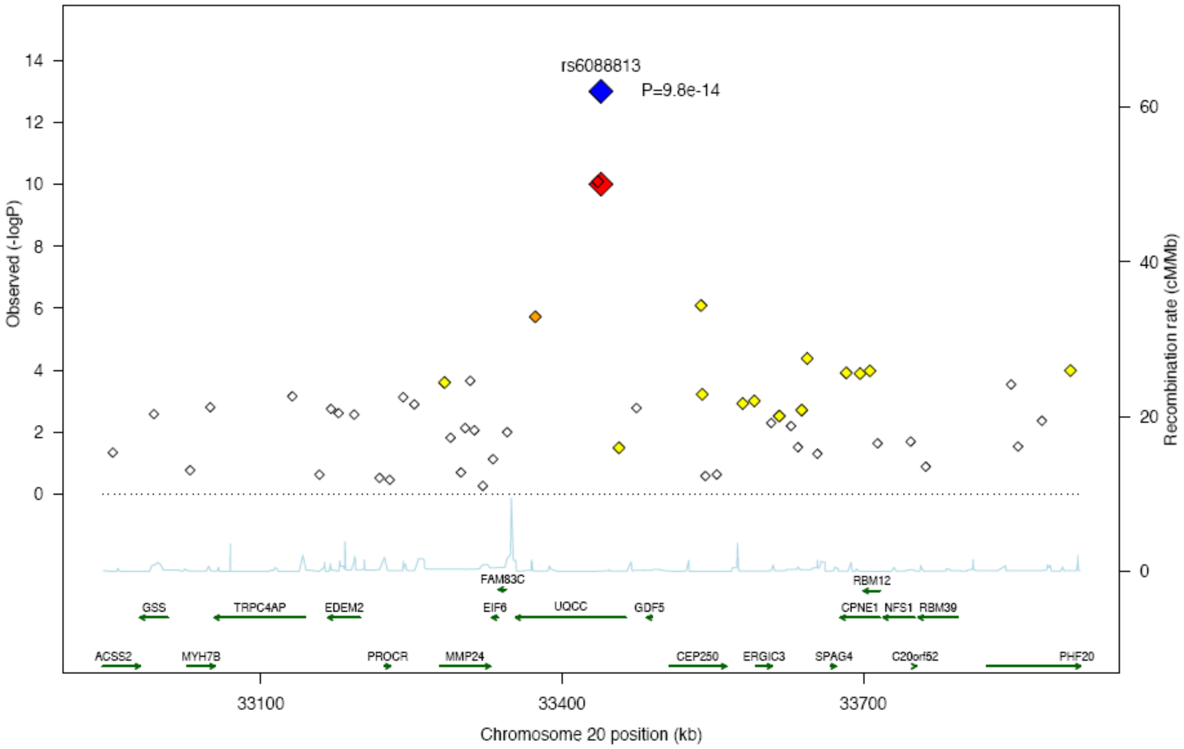
rs6088813
P=9.8e-14
Observed (-logP)
Recombination rate (cM/Mb)
Chromosome 20 position (kb)
33100
33400
33700
FAM83C
RBM12
GSS
TRPC4AP
EDEM2
EIF6
UQCC
GDF5
CPNE1
NFS1
RBM39
ACSS2
MYH7B
PROCR
MMP24
CEP250
ERGIC3
SPAG4
C20orf52
PHF20
