# Supplementary material for: Meta-Analysis of Genome-Wide Scans for Human Adult Stature Identifies Novel Loci and Associations with Measures of Skeletal Frame Size
Source: PLoS Genet. 2009 Apr 3;5(4):e1000445. doi: 10.1371/journal.pgen.1000445 (PMC2661236; doi:10.1371/journal.pgen.1000445)
Supplement: Table S1 — Distribution of height and skeletal size measurements in the cohorts included in the study. (0.09 MB PDF) [file pgen.1000445.s003.pdf]

Table S1. Distribution of height and skeletal size measurements in the cohorts included in the study

			Height			Skeletal size[3]				
	**Cohort**	**Gender**	**N**	**Age at visit (yrs) Mean (SD)**	**Height (cm) Mean (SD)**	**N[2]**	**Age at visit (yrs) Mean (SD)**	**Trunk[1] (cm) Mean (SD)**	**Hip axis (cm) Mean (SD)**	**Femur (cm) Mean (SD)**
**Discovery**	**Rotterdam Study**	**F**	3,374	69.7 (9.2)	161.4 (6.6)	1,419 /1,338	76.0 (6.5)	29.3 (1.4)	10.73 (0.6)	
		**M**	2,372	68.0 (8.0)	174.9 (6.7)	1,131 /1,030	75.2 (5.8)	31.4 (1.5)	12.34 (0.7)	
	**1958 Birth Cohort**	**F**	710	44.7	142 (6.3)					
		**M**	720	44.8	152 (6.9)					
	**TwinsUK (KCL)**	**F**	2,224	46.6 (12.2)	162.4 (6.1)					
	**EPIC Cohort**	**F**	1,129	58.7 (8.9)	161.1 (5.8)					
		**M**	988	59.2 (9)	173.8 (6.6)					
	**EPIC high-BMI cases**	**F**	617	58.9 (8.7)	160.3 (6.2)					
		**M**	477	60.1 (8.9)	173.4 (6.8)					
**Replication**	**TwinsUK (KCL_RP)**	**F**	2,294	47 (13.2)	162.5 (6.2)	2,375	48 (13.6)	42.6 (2.32)		43 (2.16)
	**Chingford**	**F**	840	54.4 (5.6)	161.7 (5.6)					
	**Chuvasha**	**F**	560	49.06 (17.08)	154.55 (6.1)	560	49.1 (17.1)	43.24 (1.48)		41.64 (2.64)
		**M**	581	47.71 (17.20)	166.34 (6.8)	581	47.7 (17.2)	46.33 (1.36)		45.10 (2.57)
	**CBR**	**F**	1,637	46.5 (12.5)	164.7 (6.6)					
		**M**	1,275	48.1 (12.1)	178.2 (6.9)					

[1] In the Rotterdam Study, trunk length was measured as the sum of individual vertebral heights. In the Chuvasha and TwinsUK collections total length of spine was used. [2] Sample numbers in the Rotterdam Study are given for vertebral size/HAL respectively. For skeletal size measurements, N= 2,375 female twins from the TwinsUK discovery and replication cohorts were analysed jointly. [3] Pearson's correlation coefficients for height-skeletal measurements are as follows. Rotterdam Study: Height-vertebral size = 0.78, Height-HAL = 0.79; TwinsUK: Height-spine = 0.59; Height-femur = 0.82; Chuvasha: Height-spine = 0.68; Height-femur = 0.83.
