# Supplementary material for: Meta-Analysis of Genome-Wide Scans for Human Adult Stature Identifies Novel Loci and Associations with Measures of Skeletal Frame Size
Source: PLoS Genet. 2009 Apr 3;5(4):e1000445. doi: 10.1371/journal.pgen.1000445 (PMC2661236; doi:10.1371/journal.pgen.1000445)
Supplement: Table S2 — Calculation of effect size for height associations by cohort. Beta and SE for height values are given in z-score units for each copy of A1 allele. For family-based cohorts (TwinsUK Discovery (KCL) and replication (KCL_RP) and Chuvasha) height z-scores were calculated in a subset of unrelated individuals. (0.15 MB PDF) [file pgen.1000445.s004.pdf]

Table S2. Calculation of effect size for height associations by cohort. Beta and SE for height values are given in z-score units for each copy of A1 allele. For family-based cohorts (TwinsUK Discovery (KCL) and replication (KCL_RP) and Chuvasha (CHU)) height z-scores were calculated in a subset of unrelated individuals.

	SNP	Locus	A1/A2	KCL Beta (SE)	RS Beta (SE)	BC58 Beta (SE)	EPIC Cohort Beta (SE)	EPIC Cases Beta (SE)	KCL_RP Beta (SE)	CHING Beta (SE)	CHU Beta (SE)	CBR Beta (SE)
$P<5x10^{-7}$	**rs11809207**	***CATSPER4***	A/G	**0.11 (0.048)**	**0.063 (0.024)**	**0.144 (0.049)**	**0.046 (0.039)**	**-0.013 (0.052)**	**0.026 (0.046)**	**0.085 (0.064)**	**0.013 (0.171)**	**0.103 (0.033)**
	**rs6763931**	***ZBTB38***	A/G	**0.008 (0.038)**	**0.078 (0.019)**	**0.052 (0.038)**	**0.089 (0.031)**	**0.1 (0.042)**	**0.067 (0.039)**	**-0.063 (0.051)**	**0.245 (0.08)**	**0.102 (0.027)**
	rs6854334	*LCORL*	T/C	0.094 (0.055)	0.074 (0.028)	0.152 (0.052)	0.114 (0.043)	0.164 (0.059)	0.028 (0.057)	0.089 (0.07)	0.119 (0.148)	
	rs6817306	*LCORL*	T/C	0.115 (0.056)	0.07 (0.028)	0.129 (0.053)	0.117 (0.044)	0.17 (0.06)	0.098 (0.057)	0.083 (0.07)	-0.133 (0.146)	
	**rs6830062**	***LCORL***	A/G	**0.093 (0.054)**	**0.077 (0.027)**	**0.150 (0.052)**	**0.111 (0.042)**	**0.159 (0.057)**	**0.102 (0.054)**	**0.06 (0.065)**	**0.104 (0.145)**	
	**rs710841**	***PRKG2***	T/C	**0.137 (0.045)**	**0.036 (0.021)**	**0.157 (0.044)**	**0.107 (0.036)**	**0.043 (0.048)**	**0.066 (0.045)**	**0.073 (0.058)**	**0.012 (0.088)**	
	rs2011962	*PRKG2*	T/G	-0.119 (0.044)	-0.043 (0.021)	-0.168 (0.043)	-0.106 (0.035)	-0.041 (0.046)	-0.065 (0.045)	-0.081 (0.058)	-0.04 (0.088)	
	**rs10472828**	***NPR3***	T/C	**-0.053 (0.039)**	**-0.06 (0.019)**	**-0.142 (0.038)**	**-0.008 (0.03)**	**-0.006 (0.041)**	**-0.097 (0.038)**	**-0.035 (0.049)**	**-0.032 (0.084)**	**-0.064 (0.026)**
	**rs10946808**	***HIST1H1D***	A/G	**0.079 (0.043)**	**0.078 (0.02)**	**0.096 (0.043)**	**0.048 (0.034)**	**0.081 (0.045)**	**0.089 (0.043)**	**0.018 (0.058)**	**0.066 (0.084)**	
	rs9358913	*HIST1H4F*	A/G	0.065 (0.044)	0.074 (0.021)	0.078 (0.044)	0.05 (0.035)	0.086 (0.046)	0.067 (0.046)			
	**rs13437082**	***HLA-B***	A/G	**-0.101 (0.044)**	**-0.105 (0.022)**	**-0.063 (0.045)**	**-0.029 (0.037)**	**-0.054 (0.047)**	**0.008 (0.043)**	**-0.052 (0.055)**	**-0.033 (0.089)**	**-0.057 (0.030)**
	rs4711269	***HLA-B***	T/C	-0.103 (0.044)	-0.104 (0.021)	-0.067 (0.044)	-0.037 (0.037)	-0.063 (0.047)	0.044 (0.044)	-0.089 (0.057)	-0.035 (0.089)	-0.058 (0.030)
	rs7742369	*HMGA1/C6orf106*	A/G	0.114 (0.05)	0.073 (0.024)	0.145 (0.049)	0.057 (0.041)	0.08 (0.056)	0.021 (0.051)	0.12 (0.066)	0.05 (0.115)	
	**rs1776897**	***HMGA1/C6orf106***	T/G	**0.126 (0.071)**	**0.13 (0.032)**	**0.193 (0.065)**	**0.09 (0.054)**	**0.064 (0.078)**	**0.057 (0.07)**	**0.233 (0.087)**	**-0.078 (0.165)**	**0.106 (0.045)**
	rs2814993	*HMGA1/C6orf106*	A/G	0.073 (0.059)	0.102 (0.026)	0.172 (0.055)	0.145 (0.043)	0.078 (0.059)	0.104 (0.054)	0.148 (0.07)	-0.019 (0.141)	0.065 (0.038)
	rs12189801	*GPR126*	T/C	0.136 (0.051)	0.103 (0.026)	0.064 (0.049)	0.064 (0.043)	-0.007 (0.056)	0.175 (0.052)	0.148 (0.068)	-0.005 (0.128)	0.072 (0.035)
	**rs6570507**	***GPR126***	T/C	**-0.086 (0.042)**	**-0.084 (0.02)**	**-0.063 (0.042)**	**-0.052 (0.033)**	**0.037 (0.046)**	**-0.143 (0.043)**		**-0.093 (0.087)**	**-0.098 (0.028)**
	**rs1182188**	***GNA12***	A/G	**0.122 (0.042)**	**0.064 (0.02)**	**0.083 (0.041)**	**0.033 (0.034)**	**0.001 (0.045)**	**0.079 (0.044)**	**0.11 (0.055)**	**0.1 (0.089)**	
	rs1182179	*GNA12*	A/G	0.118 (0.042)	0.064 (0.02)	0.081 (0.041)	0.039 (0.034)	0.007 (0.045)	0.059 (0.043)	0.083 (0.055)	0.141 (0.086)	
	**rs849141**	***JAZF1***	A/G	**0.078 (0.042)**	**0.072 (0.02)**	**0.061 (0.042)**	**0.011 (0.034)**	**0.155 (0.046)**	**0.05 (0.042)**	**0.181 (0.054)**	**-0.058 (0.107)**	**0.097 (0.029)**
	**rs2282978**	***CDK6***	A/G	**-0.087 (0.039)**	**-0.074 (0.019)**	**-0.051 (0.040)**	**-0.061 (0.033)**	**-0.069 (0.044)**	**-0.097 (0.041)**	**0.079 (0.054)**	**-0.137 (0.097)**	**-0.062 (0.028)**
	rs1480474	*HMGA2*	T/C	-0.065 (0.038)	-0.058 (0.019)	-0.127 (0.039)	-0.098 (0.031)	-0.079 (0.042)	-0.092 (0.04)	-0.03 (0.051)	0.123 (0.085)	
	**rs8756**	***HMGA2***	A/C	**-0.081 (0.038)**	**-0.062 (0.018)**	**-0.133 (0.038)**	**-0.11 (0.031)**	**-0.041 (0.043)**	**-0.095 (0.04)**	**-0.102 (0.051)**	**-0.018 (0.088)**	
	rs3118912	*DLEU7*	T/C	-0.05 (0.047)	-0.115 (0.023)	-0.082 (0.046)	-0.084 (0.038)	-0.099 (0.051)	-0.02 (0.05)		-0.125 (0.122)	
	**rs3118914**	***DLEU7***	A/C	**-0.051 (0.047)**	**-0.115 (0.023)**	**-0.082 (0.046)**	**-0.082 (0.038)**	**-0.124 (0.052)**	**-0.013 (0.049)**		**-0.13 (0.122)**	
	rs3116607	*DLEU7*	A/C	-0.037 (0.05)	-0.12 (0.025)	-0.072 (0.048)	-0.084 (0.039)	-0.103 (0.055)	-0.018 (0.05)		-0.163 (0.123)	
	rs3118916	*DLEU7*	A/G	-0.042 (0.047)	-0.102 (0.024)	-0.075 (0.046)	-0.08 (0.038)	-0.108 (0.051)	-0.029 (0.05)		-0.175 (0.121)	
	**rs910316**	***TMED10***	A/C	**0.06 (0.037)**	**0.054 (0.019)**	**0.062 (0.038)**	**0.017 (0.031)**	**0.112 (0.041)**	**0.008 (0.042)**		**0.061 (0.085)**	**0.069 (0.026)**
	rs2401171	*ADAMTSL3*	A/C	-0.09 (0.038)	-0.053 (0.018)	-0.126 (0.037)	-0.065 (0.031)	0.001 (0.041)	-0.092 (0.039)	0.033 (0.051)	-0.094 (0.082)	
	rs7183263	*ADAMTSL3*	A/C	-0.085 (0.038)	-0.055 (0.018)	-0.129 (0.037)	-0.042 (0.031)	0.012 (0.041)	-0.06 (0.039)	0.019 (0.05)	-0.086 (0.084)	-0.054 (0.027)
	**rs4842838**	***ADAMTSL3***	A/C	**0.084 (0.038)**	**0.055 (0.018)**	**0.129 (0.038)**	**0.042 (0.031)**	**-0.017 (0.041)**	**0.071 (0.039)**	**-0.012 (0.05)**	**0.102 (0.084)**	**0.055 (0.027)**
	rs4911494	*UQCC*	A/G	-0.029 (0.038)	-0.081 (0.019)	-0.114 (0.039)	-0.133 (0.032)	-0.076 (0.043)	-0.104 (0.042)	-0.107 (0.052)	-0.108 (0.082)	
	**rs6088813**	***UQCC***	A/C	**-0.03 (0.038)**	**-0.081 (0.019)**	**-0.115 (0.039)**	**-0.131 (0.032)**	**-0.072 (0.043)**	**-0.11 (0.042)**	**-0.126 (0.051)**	**-0.117 (0.082)**	
$5x10^{-7}<P<10^{-5}$	rs1812175	*HHIP*	A/G	-0.063 (0.054)	-0.042 (0.025)	-0.059 (0.052)	-0.097 (0.042)	-0.06 (0.055)	-0.139 (0.052)	0.007 (0.069)	-0.175 (0.096)	
	rs7833986	*PLAG1*	T/C	-0.083 (0.049)	-0.089 (0.025)	-0.044 (0.049)	-0.052 (0.04)	0.003 (0.054)	-0.012 (0.05)	-0.033 (0.066)	-0.123 (0.113)	0.031 (0.035)
	rs7815788	*PLAG1*	A/G	-0.046 (0.053)	-0.096 (0.029)	-0.115 (0.053)	-0.091 (0.043)	-0.035 (0.058)	-0.019 (0.056)	-0.076 (0.073)	-0.039 (0.113)	-0.058 (0.039)
	rs7871764	*WDR40A*	T/G	0.006 (0.042)	0.07 (0.02)	0.100 (0.041)	0.112 (0.034)	0.029 (0.046)				0.038 (0.038)
	rs7086883	*SH3PXD2A*	T/G	-0.027 (0.041)	-0.051 (0.02)	-0.047 (0.042)	-0.048 (0.034)	-0.155 (0.044)	-0.002 (0.043)	-0.025 (0.055)		-0.049 (0.028)
	rs3752556	*NARFL*	A/G	-0.081 (0.05)	-0.089 (0.024)	-0.012 (0.051)	-0.096 (0.041)	-0.01 (0.052)	-0.036 (0.047)	0.153 (0.064)	-0.039 (0.086)	-0.024 (0.034)
	rs12325866	*MAP3K3*	T/C	0.066 (0.043)	0.04 (0.02)	0.081 (0.042)	0.09 (0.034)	0.057 (0.046)	0.02 (0.044)	0.011 (0.058)	0 (0.097)	0.058 (0.029)
	rs6088619	*NCOA6*	T/C	-0.011 (0.053)	-0.084 (0.028)	-0.097 (0.057)	-0.151 (0.044)	-0.016 (0.062)	-0.136 (0.057)	-0.07 (0.076)	-0.25 (0.14)	-0.048 (0.039)
$P>10^{-5}$	rs955748	*WWC2*	T/C	-0.108 (0.045)	-0.055 (0.022)	-0.077 (0.044)	-0.122 (0.037)	-0.127 (0.05)	0.023 (0.05)	-0.062 (0.058)	0.027 (0.092)	-0.042 (0.030)
	rs3767141	*HSPG2*	T/C	0.039 (0.041)	0.086 (0.02)	0.078 (0.041)	-0.035 (0.033)	0.009 (0.046)	-0.06 (0.041)	0.034 (0.052)	0.022 (0.085)	0.025 (0.028)
	rs7533282	*DAB1*	A/C	-0.066 (-0.061)	-0.082 (-0.03)	-0.098 (-0.063)	-0.131 (-0.049)	-0.118 (-0.07)	0.014 (-0.06)	-0.079 (-0.075)	0.005 (-0.233)	-0.001 (-0.043)
	rs7596521	*SOCS5*	A/G	-0.074 (-0.043)	-0.067 (-0.021)	-0.025 (-0.043)	-0.068 (-0.034)	-0.077 (-0.045)	-0.005 (-0.043)	0.003 (-0.056)	-0.05 (-0.093)	0.015 (-0.030)
	rs753628	*FAM43A/LSG1*	T/C	0.085 (0.039)	0.041 (0.019)	0.119 (0.039)	0.042 (0.031)	0.04 (0.044)	0.019 (0.04)	-0.1 (0.051)	0.011 (0.083)	-0.003 (0.027)
	rs2714357	*RREB1*	A/C	0.013 (0.041)	0.057 (0.019)	0.108 (0.040)	0.057 (0.033)	0.043 (0.044)	0.077 (0.041)	0.043 (0.054)	-0.118 (0.087)	-0.027 (0.028)
	rs742106	*DTNBP1*	A/G	0.098 (0.04)	0.061 (0.019)	0.060 (0.040)	0.048 (0.032)	0.074 (0.044)	-0.043 (0.042)	-0.039 (0.055)	-0.093 (0.084)	0.016 (0.027)
	rs3131296	*NOTCH4*	A/G	0.085 (0.055)	0.096 (0.026)	0.105 (0.053)	0.013 (0.044)	0.028 (0.059)	0.001 (0.052)	0.017 (0.071)	0.197 (0.142)	0.066 (0.037)
	rs1523632	*AGR2*	T/C	0.082 (0.038)	0.062 (0.019)	0.054 (0.039)	0.045 (0.031)	-0.011 (0.041)	-0.033 (0.045)	0.102 (0.049)		
	rs3125945	*NLGN3*	T/C	0.069 (0.039)	0.071 (0.016)	-0.032 (0.031)	0.026 (0.026)	0.041 (0.036)		-0.069 (0.05)		0.005 (0.022)
	rs1402078	*NAP1L3*	T/C	-0.139 (0.06)	-0.065 (0.024)	-0.030 (0.046)	-0.063 (0.038)	-0.071 (0.055)		-0.105 (0.076)		0.051 (0.034)
