# Supplementary material for: Meta-Analysis of Genome-Wide Scans for Human Adult Stature Identifies Novel Loci and Associations with Measures of Skeletal Frame Size
Source: PLoS Genet. 2009 Apr 3;5(4):e1000445. doi: 10.1371/journal.pgen.1000445 (PMC2661236; doi:10.1371/journal.pgen.1000445)
Supplement: Table S3 — Gender-specific associations at the 17 validated height loci. (0.11 MB PDF) [file pgen.1000445.s005.pdf]

Table S3. Gender-specific associations at the 17 validated height loci

SNP	Locus	Females (n=11,721)[1]		Males (n=5,107)[1]		Males vs females[2]		
		Beta (SE)	P-value	Beta (SE)	P-value	Q (1 d.f.)	Q P-value	I^2
**rs11809207**	*CATSPER4*	0.046 (0.009)	$4.7x10^{-6}$	0.027 (0.025)	0.102	0.01	0.91	0
**rs6763931**	*ZBTB38*	0.024 (0.007)	$1.4x10^{-7}$	0.046 (0.02)	$9.8x10^{-4}$	1.81	0.18	45
**rs6854334**	*LCORL*	0.038 (0.01)	$8.4x10^{-5}$	0.116 (0.033)	$1.7x10^{-4}$	0.21	0.64	0
**rs6817306**	*LCORL*	0.039 (0.011)	$6.3x10^{-6}$	0.102 (0.034)	0.0015	0.04	0.83	0
**rs6830062**	*LCORL*	0.042 (0.01)	$4.3x10^{-6}$	0.11 (0.032)	$2.0x10^{-4}$	0.07	0.78	0
**rs710841**	*PRKG2*	0.039 (0.009)	$5.6x10^{-6}$	0.074 (0.026)	$5.5x10^{-4}$	1.62	0.20	38
**rs2011962**	*PRKG2*	-0.04 (0.009)	$1.7x10^{-6}$	-0.07 (0.026)	$7.0x10^{-4}$	2.28	0.13	56
**rs10472828**	*NPR3*	-0.016 (0.007)	$1.5x10^{-3}$	-0.037 (0.019)	$2.4 x10^{-3}$	0.89	0.34	0
**rs10946808**	*HIST1H1D*	0.031 (0.008)	$1.4x10^{-9}$	0.073 (0.025)	$1.6x10^{-4}$	0.84	0.36	0
**rs9358913**	*HIST1H4F*	0.028 (0.009)	$1.5x10^{-7}$	0.06 (0.026)	0.010	1.07	0.30	6
**rs13437082**	*HLA-B*	-0.03 (0.009)	$4.1x10^{-6}$	-0.047 (0.023)	0.011	0.73	0.39	0
**rs4711269**	*HLA-B*	-0.03 (0.009)	$6.1x10^{-6}$	-0.048 (0.023)	0.015	1.04	0.31	4
**rs7742369**	*HMGA1/ C6orf106*	0.039 (0.01)	$9.1x10^{-7}$	0.064 (0.029)	0.008	0.01	0.94	0
**rs1776897**	*HMGA1/ C6orf106*	0.05 (0.013)	$2.8x10^{-7}$	0.092 (0.034)	0.0014	1.03	0.31	3
**rs2814993**	*HMGA1/ C6orf106*	0.049 (0.011)	$8.9x10^{-7}$	0.092 (0.028)	$1.5x10^{-4}$	0.04	0.84	0
**rs12189801**	*GPR126*	0.029 (0.01)	$1.0x10^{-7}$	0.071 (0.028)	0.0026	0.95	0.33	0
**rs6570507**	*GPR126*	-0.026 (0.008)	$1.0x10^{-6}$	-0.055 (0.022)	0.0019	0.48	0.49	0
**rs1182188**	*GNA12*	0.02 (0.008)	$2.3x10^{-8}$	0.032 (0.025)	0.0064	1.18	0.28	15
**rs1182179**	*GNA12*	0.019 (0.008)	$5.0x10^{-8}$	0.036 (0.025)	0.003	1.12	0.29	11
**rs849141**	*JAZF1*	0.024 (0.008)	$1.4x10^{-7}$	0.041 (0.022)	0.0076	0.30	0.59	0
**rs2282978**	*CDK6*	-0.025 (0.008)	$3.6x10^{-6}$	-0.046 (0.021)	0.0063	0.49	0.48	0
**rs1480474**	*HMGA2*	-0.039 (0.008)	$4.6x10^{-9}$	-0.065 (0.023)	$1.8x10^{-4}$	0.61	0.43	0
**rs8756**	*HMGA2*	-0.039 (0.007)	$3.3x10^{-11}$	-0.07 (0.023)	$4.2x10^{-5}$	0.50	0.48	0
**rs3118912**	*DLEU7*	-0.023 (0.009)	$2.5x10^{-5}$	-0.099 (0.029)	$5.7x10^{-5}$	1.31	0.25	23
**rs3118914**	*DLEU7*	-0.023 (0.009)	$2.0x10^{-5}$	-0.101 (0.029)	$5.4x10^{-5}$	1.52	0.22	34
**rs3116607**	*DLEU7*	-0.022 (0.01)	$3.2x10^{-5}$	-0.095 (0.03)	$2.1x10^{-4}$	1.37	0.24	27
**rs3118916**	*DLEU7*	-0.024 (0.009)	$2.4x10^{-5}$	-0.089 (0.029)	$1.6x10^{-4}$	1.54	0.22	35
**rs910316**	*TMED10*	0.022 (0.007)	$6.7x10^{-5}$	0.038 (0.02)	0.0072	0.65	0.42	0
**rs2401171**	*ADAMTSL3*	-0.032 (0.007)	$1.0x10^{-8}$	-0.003 (0.023)	0.222	6.89	0.01	85
**rs7183263**	*ADAMTSL3*	-0.026 (0.007)	$9.7x10^{-8}$	0.002 (0.02)	0.215	9.37	0.002	89
**rs4842838**	*ADAMTSL3*	-0.027 (0.007)	$3.6x10^{-8}$	0.004 (0.02)	0.251	9.39	0.002	89
**rs4911494**	*UQCC*	-0.035 (0.008)	$5.0x10^{-9}$	-0.102 (0.023)	$1.9x10^{-6}$	1.37	0.24	27
**rs6088813**	*UQCC*	-0.035 (0.008)	$3.7x10^{-9}$	-0.101 (0.023)	$2.1x10^{-6}$	1.31	0.25	24

[1] Meta-analysis P-values for the female and male samples were calculated from study-specific best analysis P-values weighted by sample size. Betas and SE were calculated using inverse variance meta-analysis for height values normalised to z-scores. For family-based cohorts, such values were calculated in a subset of unrelated individuals (n = 1,381 for TwinsUK discovery and n = 1,403 for TwinsUK replication).
[2] Rotterdam Study only
