# Supplementary material for: Meta-Analysis of Genome-Wide Scans for Human Adult Stature Identifies Novel Loci and Associations with Measures of Skeletal Frame Size
Source: PLoS Genet. 2009 Apr 3;5(4):e1000445. doi: 10.1371/journal.pgen.1000445 (PMC2661236; doi:10.1371/journal.pgen.1000445)
Supplement: Table S4 — Association of height loci with femur length. For each locus, the meta-analysis P-value was calculated for best analysis on two cohorts (TwinsUK, N = 2,364 and Chuvasha, N = 1,141). Univariate and conditional analyses were carried out on a subset of unrelated TwinsUK samples with available height and femur length. (0.12 MB PDF) [file pgen.1000445.s006.pdf]

Table S4. Association of validated height loci with femur length. For each locus, the meta-analysis P-value was calculated for best analysis on two cohorts (TwinsUK, N=2,364 and Chuvasha, N=1,141). Univariate and conditional analyses were carried out on a subset of 1,235 unrelated TwinsUK samples with available height and femur length from gender- and age-adjusted z-scores.

SNP	NCBI gene	A1/A2	All samples (n=3,505)			TwinsUK[2]			
						Univariate		Height-adjusted	
			Zscore	P-value	% variance	Beta (SE)	P-value	Beta (SE)	P-value
**rs11809207**	*CATSPER4*	A/G	1.533	0.125	0.096	0.06 (0.051)	0.236	0.01 (0.029)	0.724
**rs6763931**	*ZBTB38*	A/G	1.503	0.133	0.125	0.052 (0.039)	0.183	-0.002 (0.022)	0.931
**rs6854334**	*LCORL*	T/C	1.503	0.133	0.068	0.064 (0.059)	0.280	0.006 (0.034)	0.863
**rs6817306**	*LCORL*	T/C	1.17	0.242	0.188	0.094 (0.06)	0.117	0.010 (0.034)	0.772
**rs6830062**	*LCORL*	T/C	0.48	0.631	0.326	0.119 (0.057)	0.038	0.023 (0.033)	0.49
**rs710841**	*PRKG2*	T/C	4.223	2.4x10^{-5}	0.451	0.08 (0.046)	0.086	0.016 (0.026)	0.542
**rs2011962**	*PRKG2*	A/C	-3.592	3.3x10^{-4}	0.336	-0.071 (0.046)	0.122	-0.020 (0.026)	0.44
**rs10472828**	*NPR3*	T/C	-0.473	0.637	0.203	-0.082 (0.041)	0.044	-0.005 (0.023)	0.831
**rs10946808**	*HIST1H1D*	A/G	4.511	6.4x10^{-6}	0.776	0.143 (0.045)	0.002	0.061 (0.026)	0.018
**rs9358913**	*HIST1H4F*	A/G	3.968	7.2x10^{-5}	0.763	0.138 (0.047)	0.003	0.080 (0.026)	0.002
**rs13437082**	*HLA-B*	T/C	-1.644	0.100	0.026	-0.027 (0.047)	0.557	4.8x10^{-4} (0.026)	0.986
**rs4711269**	*HLA-B*	T/C	-0.919	0.358	0.016	-0.024 (0.047)	0.614	0.006 (0.026)	0.833
**rs7742369**	*HMGA1/C6orf106*	A/G	-1.322	0.186	0.191	-0.079 (0.053)	0.136	-0.034 (0.003)	0.257
**rs1776897**	*HMGA1/C6orf106*	T/G	-1.274	0.203	0.178	-0.081 (0.07)	0.245	-0.014 (0.040)	0.719
**rs2814993**	*HMGA1/C6orf106*	A/G	1.14	0.254	0.010	0.022 (0.058)	0.708	-0.046 (0.033)	0.162
**rs12189801**	*GPR126*	T/C	1.386	0.166	0.103	0.086 (0.055)	0.120	0.005 (0.031)	0.872
**rs6570507**	*GPR126*	A/G	-0.985	0.325	0.004	-0.037 (0.044)	0.402	0.029 (0.025)	0.258
**rs1182188**	*GNA12*	T/C	2.412	0.016	0.563	0.094 (0.044)	0.032	-0.007 (0.025)	0.779
**rs1182179**	*GNA12*	A/G	-0.79	0.430	0.441	0.085 (0.044)	0.055	-0.014 (0.025)	0.579
**rs849141**	*JAZF1*	A/G	1.888	0.059	0.448	0.108 (0.043)	0.013	0.036 (0.025)	0.151
**rs2282978**	*CDK6*	T/C	-3.479	0.001	0.271	-0.068 (0.043)	0.111	-0.001 (0.024)	0.958
**rs1480474**	*HMGA2*	A/G	0.613	0.540	0.151	0.046 (0.041)	0.265	0.001 (0.023)	0.967
**rs8756**	*HMGA2*	A/C	-2.363	0.018	0.119	-0.045 (0.04)	0.258	0.007 (0.023)	0.769
**rs3118912**	*DLEU7*	T/C	-0.199	0.842	0.009	-0.001 (0.049)	0.987	0.003 (0.028)	0.921
**rs3118914**	*DLEU7*	T/G	-1.976	0.048	0.012	-0.003 (0.049)	0.956	0.001 (0.028)	0.981
**rs3116607**	*DLEU7*	A/C	-1.018	0.309	-	0.025 (0.052)	0.626	0.002 (0.029)	0.948
**rs3118916**	*DLEU7*	A/G	-1.855	0.064	0.031	-0.015 (0.05)	0.759	4.4x10^{-4} (0.028)	0.988
**rs910316**	*TMED10*	A/C	0.529	0.597	0.032	0.047 (0.042)	0.270	0.027 (0.024)	0.258
**rs2401171**	*ADAMTSL3*	T/G	0.185	0.854	0.988	-0.13 (0.041)	0.001	-0.061 (0.023)	0.008
**rs7183263**	*ADAMTSL3*	T/G	-2.642	0.008	0.765	-0.118 (0.04)	0.004	-0.05 (0.023)	0.03
**rs4842838**	*ADAMTSL3*	T/G	0.449	0.653	0.902	0.130 (0.04)	0.001	0.054 (0.023)	0.018
**rs4911494**	*UQCC*	T/C	0.211	0.833	0.425	-0.087 (0.041)	0.034	0.023 (0.024)	0.335
**rs6088813**	*UQCC*	A/C	-0.312	0.755	0.350	-0.078 (0.042)	0.062	0.022 (0.024)	0.346
